# Design of a multi-epitope recombinant BCG vaccine targeting Brucella OMP31, LptE and VirB2 in immunoinformatics approaches

**DOI:** 10.1371/journal.pone.0334843

**Published:** 2025-11-06

**Authors:** Chuang Li, Yuejie Zhu, Xingxing Qi, Zhenglong Chai, Jiarui Luo, Kaiyu Shang, Tingting Tian, Huidong Shi, Mingzhe Li, Ruixue Xu, Fuling Pu, Junyu Kuang, Fengbo Zhang

**Affiliations:** 1 State Key Laboratory of Pathogenesis, Prevention and Treatment of High Incidence Diseases in Central Asia, The First Affiliated Hospital of Xinjiang Medical University, Urumqi, China; 2 Reproductive Medicine Center, The First Affiliated Hospital of Xinjiang Medical University, Urumqi, China; 3 Post-doctoral Research station of the Clinical Medicine, The First Affiliated Hospital of Xinjiang Medical University, Urumqi, China; 4 Department of Medicine, The First Clinical Medical College, Xinjiang Medical University, Urumqi, China; Albert Einstein College of Medicine, UNITED STATES OF AMERICA

## Abstract

Brucellosis, caused by the intracellular pathogen *Brucella*, remains a significant health challenge, alongside substantial economic impacts on livestock industries. Despite antibiotic treatments, the absence of licensed human vaccines necessitates innovative preventive strategies. In this study, we employed reverse vaccinology to design a novel multi-epitope vaccine (MEV) targeting *Brucella melitensis*. Three immunogenic proteins—outer membrane protein OMP31, LPS assembly protein LptE, and the type IV secretion system protein VirB2—were selected as vaccine candidates. Comprehensive bioinformatics analysis identified six cytotoxic T lymphocyte (CTL) epitopes, nine helper T lymphocyte (HTL) epitopes, seven linear B-cell epitopes, and five conformational B-cell epitopes. The incorporation of molecular adjuvants (cholera toxin B subunit and PADRE) served to further enhance the immunogenicity of the vaccine. Given that Brucella is an intracellular parasite, TAT cell-penetrating peptides were added to further enhance the intracellular delivery of MEV. The constructed MEV has been shown to have excellent antigenicity (VaxiJen score >0.8), stability (instability index <40), solubility (Protein-Sol score: 0.87) and hydrophilicity (GRAVY index: −0.319), and is non-allergenic. Structural optimization, including disulfide bond engineering (11 pairs of residues), improved molecular stability, with molecular docking and dynamics simulations confirming robust interactions with immune cell receptors (docking score: −311.85). Using SnapGene 7.1.2, we performed *in silico* cloning simulation of the codon-optimized multi-epitope vaccine (MEV) sequence into the pMV261 shuttle vector, generating a recombinant BCG (rBCG) construct. Immunoinformatics simulations (C-ImmSim) demonstrated potent immune activation, with significant increases in cytotoxic T cells (1050 cells/mm³), memory helper T cells (1150 cells/mm³), and IFN-γ production (2 × 10^6 ng/ml), alongside sustained IgG/IgM titers over 350 days(1 × 10^5 cells/mm^3^) . Furthermore, the recombinant BCG multi-epitope Brucella vaccine, developed through bioinformatics approaches, demonstrates promising characteristics and immunogenicity. Nevertheless, its immunological efficacy requires to further experimental validation.

## 1. Introduction

Brucella, a Gram-negative intracellular pathogen, represents a significant public health concern as it causes severe zoonotic infections transmitted through aerosol inhalation, ingestion of contaminated food, or direct contact with infected animals [1]. This pathogen demonstrates remarkable immune evasion capabilities, leading to chronic infections characterized by fever, fatigue, arthralgia, and potentially severe arthro-skeletal and neurological complications [2,3]. Beyond its clinical impact, brucellosis imposes substantial economic burdens on global livestock industries [3]. Although combination antibiotic therapy remains the primary treatment strategy, the emergence of drug-resistant Brucella strains has complicated therapeutic interventions [4]. These challenges have intensified the demand for novel, safe, and effective vaccines, particularly for deployment in endemic regions [5].

This study aimed to design a novel multi-epitope Brucella vaccine using reverse vaccinology, hypothesizing that integrating OMP31, LptE, and VirB2 epitopes with molecular adjuvants in an rBCG vector would elicit potent and durable immune responses against brucellosis. Our approach focused on the Brucella outer membrane protein Omp31, a well-characterized vaccine antigen. The complex pathogenesis of Brucella, mediated by virulence factors including lipopolysaccharide (LPS), the type IV secretion system (T4SS), and the BvrR/BvrS regulatory system, contributes to its persistent infections [6]. We specifically targeted the lipopolysaccharide transporter protein LptE, essential for LPS assembly on the bacterial outer membrane [7], and VirB2, a critical component of the T4SS apparatus that facilitates intracellular survival and replication [8,9]. Bioinformatic analysis revealed that these three candidate proteins exhibited superior antigenic properties, with antigenicity scores exceeding the threshold value of 0.4. Importantly, they demonstrated favorable characteristics including non-allergenicity, non-toxicity, structural stability, and membrane localization, making them ideal candidates for vaccine development against Brucella infections.

Signal peptide analysis of the three candidate proteins was conducted using SignalP 6.0 and LIPOP 1.0, followed by removal of identified signal sequences. One of the most important applications of immunoinformatics is the prediction of multiple specific epitopes for B-cell recognition and T-cell recognition of MHC class I and II molecules [10]. Immunoinformatics-driven epitope prediction was systematically performed using IEDB, NetMHCIIpan, and ABCpred platforms to identify MHC class I/II-restricted T-cell epitopes and B-cell linear/conformational epitopes. The subdominant epitopes were subjected to rigorous screening on the basis of epitope score, immunogenicity, allergenicity, instability and toxicity. In order to address the potential immunogenicity limitations of multi-epitope vaccines, molecular adjuvants (CTB and PADRE) were integrated [11], and intracellular delivery of the vaccine was enhanced using TAT cell-penetrating peptides. Consequently, a multi-epitope vaccine was constructed by linking antigenic epitopes and molecular adjuvants using EAAK, AAY, GPGPG and KK connectors. The biophysical properties of the vaccine construct, including antigenicity (VaxiJen score >0.8), allergenicity (AllergenFP prediction: non-allergenic), stability (instability index <40), and solubility (Protein-Sol score >0.7), were computationally validated. Structural characterization of vaccine was performed using SOMPA for secondary structure prediction and Robetta for tertiary structure modeling. Disulfide bond engineering (DsbServer) introduced stabilising bonds (energy <2.0 kcal/mol) on 11 pairs of residues, enhancing the vaccine structural integrity. Molecular docking (HDOCK) and molecular dynamics simulations (GROMACS) confirmed a stable vaccine-TLR4 interaction (docking score: −311.85) with good deformability and residue mobility characteristics. Building on the established safety and efficacy of Bacillus Calmette-Guérin (BCG) as a vaccine vector [12], we engineered a recombinant BCG (rBCG) expressing the multi-epitope vaccine (MEV). The rBCG platform offers distinct advantages, including proven safety, non-specific immune stimulation, and multivalent potential, as demonstrated in HIV Ⅰ-BCG [13] and RSV-BCG [14] vaccine development. Successful computer-simulated cloning benefited from using ExpOptimizer for codon optimization and SnapGene for primer design, MEV DNA sequence amplification, selection of pMV261-BCG shuttle plasmid, and insertion of amplified vaccine DNA sequence into the plasmid. This ultimately successfully simulated the construction of recombinant BCG vaccine plasmid in SnapGene software. Immunoinformatics simulation (C-ImmSim) predicted robust immune activation, with significant increases in cytotoxic T cells (1050 cells/mm³), memory helper T cells (1150 cells/mm³), and IFN-γ production (2 × 10^6 ng/ml), alongside sustained IgG/IgM titers. These results demonstrate that the rBCG-MEV construct meets critical vaccine design criteria, elicits potent immune responses, and represents a safe and promising candidate for *Brucella melitensis* prevention. This study offers novel development strategies for future brucellosis vaccines. However, based on computer-based simulations, its immunogenicity and practical efficacy still require experimental validation.

## 2. Materials and methods

### 2.1 Bioinformatic analysis of target antigens

**Fig 1:**
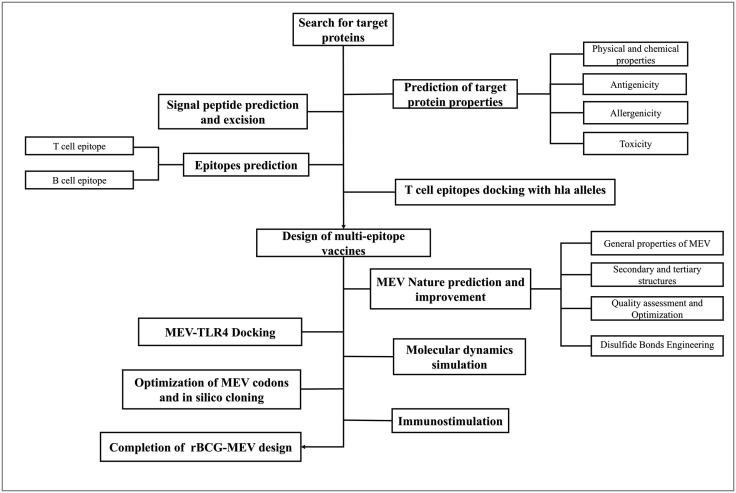
The flow of vaccine design and evaluation.

Amino acid sequences of *Brucella* outer membrane protein OMP31 (UniProt ID:C0RKW7, https://www.uniprot.org/uniprotkb/C0RKW7/entry), lipopolysaccharide transporter LptE (UniProt ID:C0RF57, https://www.uniprot.org/uniprotkb/C0RF57/entry), and type IV secretion system protein VirB2 (UniProt ID:C0RK20, https://www.uniprot.org/uniprotkb/C0RK20/entry) were retrieved from the UniProt database. These three proteins play important roles in Brucella survival, preventing host phagocytosis, and intracellular infection [6]. The relevant physicochemical properties of proteins are key in determining whether a protein can be used as a vaccine. ProtParam server utilizes information from the SWISS-PROT database to predict the physical and chemical properties of proteins [15]. Key physicochemical parameters including amino acid count, molecular formula, theoretical isoelectric point (pI), instability index, and grand average of hydropathicity (GRAVY) were analyzed using the ProtParam server (http://web.expasy.org/protparam/). The VaxiJen 2.0 derives models for predicting the antigenicity of whole proteins using bacterial, viral, and tumor protein datasets based on the automated cross-covariance (ACC) transformations of protein sequences [16,17]. Antigenicity prediction was performed through VaxiJen 2.0 (https://www.ddg-pharmfac.net/vaxijen/VaxiJen/VaxiJen.html) with a bacterial pathogen model threshold of 0.4. Toxicity profiles were assessed using ToxinPred, while allergenicity potential was evaluated via AllergenFP v1.1(https://www.ddg-pharmfac.net/AllergenFP/) . Allergen FP is a descriptor-based fingerprinting method that correctly identified 88% of samples with a Matthews correlation coefficient of 0.759 without comparison and has been applied to the identification of allergens and non-allergens [18]. The sequence similarity of candidate antigens among Brucella pathogenic species was analyzed using Clustal Omega and visualized using Jalview software [19]. A systematic workflow detailing multi-epitope vaccine (MEV) design and evaluation is presented in Fig 1.

### 2.2 Prediction of protein signal peptides

SignalP 6.0 (https://services.healthtech.dtu.dk/services/SignalP-6.0/) is a machine learning model that can detect all types of signal peptides and is suitable for metagenomic data [20]. LipoP (https://www.ddg-pharmfac.net/AllergenFP/) is a method for predicting signal peptides of lipoproteins in Gram-negative bacteria, which correctly predicts 96.8% of lipoproteins with only 0.3% false positives [21]. As these non-essential structural elements may interfere with epitope presentation in multi-epitope vaccines, a consensus approach integrating both prediction tools was implemented to identify and excise signal peptide regions. N-terminal sequences demonstrating positive signal peptide characteristics in either analysis were subsequently excluded from vaccine construct design.

### 2.3 T-cell epitope prediction

The recognition of antigenic epitopes by immune cells represents a critical step in immune activation, where antigen-presenting cells process immune proteins into small fragments that are recognized by T cells through MHC-epitope-TCR interactions, with CD4 + T cells recognizing helper T lymphocyte (HTL) epitopes to differentiate into either helper T lymphocytes (HTL) or regulatory T cells (Treg), and CD8 + T cells recognizing cytotoxic T lymphocyte (CTL) epitopes to differentiate into cytotoxic T lymphocytes (CTL). Given the high incidence of brucellosis in the Xinjiang region, we adopted the following criteria to select high-frequency alleles: ① Uyghur frequency >7% (to ensure population coverage); ② presence of strongly linked haplotypes (to enhance epitope presentation efficiency); ③ Low-frequency alleles (<1%) were excluded due to marginal effects. Ultimately, high-frequency HLA alleles were included, including HLA-A1101 (13.46%), HLA-A0201 (12.50%), HLA-A*0301 (10.10%), HLA-DRB1 * 0701 (16.35%), HLA-DRB1 * 1501 (8.65%), and HLA-DRB1 * 0301 (7.69%) [22,23]. NetMHCpan-4.1 integrates binding affinity and mass spectrometry elution to enhance the predictive performance of ligand data, achieving state-of-the-art predictive performance and surpassing competitors [24]. CTL epitopes were predicted using the IEDB MHC-I binding tool (http://tools.iedb.org/mhci/) with the NetMHCpan 4.1 EL method, selecting the three HLA-A alleles, while maintaining all other software parameters at default settings. In large-scale benchmark testing, NetMHCIIpan-4.0 demonstrated superior performance to current state-of-the-art predictors in ligand and CD4 + T cell epitope prediction, making it a powerful tool for predicting T cell epitopes and developing personalized immunotherapies [25]. HTL epitopes were predicted using the NetMHCIIpan-4.1 online software (https://services.healthtech.dtu.dk/services/NetMHCpan-4.1/), selecting the three HLA-DRB1 alleles and using default software parameters. Predicted epitopes were subsequently validated through antigenicity analysis using VaxiJen 2.0, allergenicity detection using AllergenFP v1.1, toxicity prediction using ToxinPred (https://webs.iiitd.edu.in/raghava/toxinpred/design.php), and stability analysis using ProtParam, with the top three sequences exhibiting the highest scores, no allergenicity, high immunogenicity, and no toxicity selected as the dominant T cell epitopes for vaccine construction.

### 2.4 B-cell epitope prediction

B cell activation involves the recognition of both linear and conformational epitopes, triggering antibody production, with linear epitopes predicted through consensus analysis using ABCpred (https://webs.iiitd.edu.in/raghava/abcpred/ABC_submission.html) and IEDB B cell epitope tool (http://tools.iedb.org/bcell/) , where overlapping sequences identified by both platforms were selected as dominant linear epitopes. BepiPred-2.0 is based on a random forest algorithm trained on epitopes annotated in antibody-antigen protein structures, and this method has been shown to outperform other available tools in sequence-based epitope prediction [26]. ElliPro was tested on a benchmark dataset for discontinuous epitope inference based on antibody-protein complex three-dimensional structures. Compared with other structural methods available for epitope prediction, ElliPro performed best and is considered a practical research tool for identifying antibody epitopes in protein antigens [27]. The conformational epitopes were analyzed via IEDB’s Ellipro (http://tools.iedb.org/ellipro/), retaining the top 10 scoring peptides based on ElliPro score thresholds, and all candidate epitopes underwent comprehensive profiling using VaxiJen 2.0 (threshold >0.4) for antigenicity, AllergenFP v1.1 for allergenicity, ProtParam (instability index <40) for physicochemical stability, and ToxinPred for toxicity, with final epitope selection prioritizing sequences demonstrating concurrent antigenicity (VaxiJen score >0.8), non-allergenicity, structural stability, and non-toxicity for multi-epitope vaccine (MEV) assembly.

### 2.5 Molecular docking of CTL and HTL epitopes with HLA alleles

The stable binding of T-cell epitopes to HLA molecules is essential for effective antigen presentation by antigen-presenting cells (APCs) [28], which was evaluated using the HDOCK server (http://hdock.phys.hust.edu.cn/) for molecular docking analysis. HDOCK is a new web server based on a hybrid docking algorithm (template modeling and free docking) that combines HDOCK and template-based modeling results to further improve the server’s predictive capabilities by ranking template-based models [29–31].The HLA class I (HLA-A*02:01) and HLA class II (HLA-DRB1 * 01:01) alleles were selected to dock with predicted CTL and HTL epitopes, respectively, revealing specific interactions between the alleles and T-cell epitopes based on binding energy and intermolecular contacts [32].

### 2.6 Multi-epitope vaccine construction

The multi-epitope vaccine (MEV) was constructed by integrating selected dominant T and B cell antigenic epitopes, with adjuvants incorporated to enhance immunogenicity. To address the potential reduced immunogenicity of multi-epitope vaccines compared to live attenuated vaccines, cholera toxin B (CTB) (AIE88420.1) and Pan HLA-DR epitopes (PADRE) were added to the N-terminus of the vaccine construct [33]. The CTB was connected to PADRE via an EAAK linker, while PADRE was linked to the CTL epitope using an AAY linker. CTL epitopes were sequentially connected to HTL epitopes via GPGPG linkers, and HTL epitopes were linked to B-cell epitopes using KK linkers [34]. Given Brucella’s intracellular parasitism, which complicates its elimination, the TAT sequence (TGALLAAGAAA) was incorporated at the C-terminus of the vaccine construct via a KK linker to enhance cellular penetration and vaccine efficacy [35,36]. The final construct, designated as the Brucella multi-epitope vaccine (MEV), was optimized for both immunogenicity and intracellular delivery.

### 2.7 Prediction of physicochemical and functional properties of MEV

The multi-epitope vaccine (MEV) was comprehensively characterized using computational tools to evaluate its physicochemical and immunological properties. The ProtParam tool (http://web.expasy.org/protparam/) was employed to predict key physicochemical parameters, including amino acid composition, molecular weight, theoretical isoelectric point (pI), instability index, grand average of hydropathicity (GRAVY) and aliphatic index, where an instability index <40 indicated structural stability and a negative GRAVY value confirmed hydrophilic properties. A higher aliphatic index of the vaccine protein correlates with enhanced thermostability [37].Antigenicity was assessed using the VaxiJen2.0 server (https://www.ddg-pharmfac.net/vaxijen/VaxiJen/VaxiJen.html) with a threshold >0.4, ensuring robust immunogenicity. Allergenicity was analyzed via AllergenFP v1.1 (https://www.ddg-pharmfac.net/AllergenFP/) to confirm non-allergenic characteristics, while solubility was predicted using the Protein-Sol server (http://scratch.proteomics.ics.uci.edu/) with a threshold >0.5, ensuring favorable solubility for vaccine formulation and administration. These analyses collectively validated the MEV’s stability, immunogenicity, safety, and solubility, supporting its potential as a viable vaccine candidate.

### 2.8 Secondary and tertiary structure prediction of MEV

The secondary structure of the vaccine protein was predicted using the Self-Optimizing Prediction Method (SOPM) implemented in the SOMPA online tool (https://npsa.lyon.inserm.fr/cgi-bin/npsa_automat.pl?page=/NPSA/npsa_sopma.html), which enhances prediction accuracy through consistent multiple comparisons [38]. Subsequently, the tertiary structure was modeled using RoseTTAFold via the Robetta server (https://robetta.bakerlab.org/), a state-of-the-art approach that employs a 3-track neural network to achieve ultra-high accuracy in protein structure and interaction prediction [39]. These computational analyses provided critical insights into the vaccine protein’s structural organization, supporting its functional characterization and optimization.

### 2.9 Tertiary structure of MEV optimization and assessment

The tertiary structure of MEV was refined using the GalaxyRefine web server (https://galaxy.seoklab.org/cgi-bin/submit.cgi?type=REFINE), which employs side chain reconstruction and molecular dynamics simulations for side chain rearrangement and overall structural relaxation, thereby enhancing both global and local structural quality [40,41]. Structural validation was performed through multiple computational approaches. The Ramachandran plot analysis, conducted using the Procheck Web Server (https://saves.mbi.ucla.edu/), was utilized to evaluate the psi and phi dihedral angles, providing an assessment of the overall stereochemical quality of the vaccine structure [42]. Additionally, the three-dimensional structure of the designed vaccine construct was validated using ProSA-web (https://prosa.services.came.sbg.ac.at/prosa.php) for potential errors in 3D structure and ERRAT for statistical analysis of non-bonded atomic interactions [43]. The ProSA program (protein structure analysis) is a mature tool with a large user base that is frequently used for refining and validating experimental protein structures, as well as for structure prediction and modeling [44].

### 2.10 Disulfide bonds engineering

The incorporation of disulfide bonds into the vaccine protein was performed to enhance protein folding and improve overall structural stability. Disulfide by Design 2.0 (DbD2) significantly surpasses the original program in terms of functionality, visualization, and analysis capabilities, helping to identify potential disulfide bonds that not only may form but are also expected to improve protein thermal stability [45]. Disulfide bond engineering was conducted using the Disulfide by Design server v2.12 (http://cptweb.cpt.wayne.edu/DbD2/), with default parameters maintained for both the Cα-Cβ-Sγ angle and χ3 value. Potential disulfide bonds were identified based on stringent selection criteria, including an energy threshold of <2.0 kcal/mol and χ3 values ranging from −87° to +97° (±30°) [46]. Following disulfide bond construction, the structural quality of the modified vaccine protein was validated using ProSA-web and ERRAT analyses to ensure maintenance of structural integrity.

### 2.11 Protein-protein docking

Pathogen recognition represents a critical initial step in initiating an effective immune response during infection. Toll-like receptors (TLRs), particularly TLR4, play a pivotal role in immune priming through their capacity to recognize diverse molecular patterns associated with invading pathogens [47,48]. Molecular docking analysis was performed using the HDOCK server (http://hdock.phys.hust.edu.cn/) to evaluate the binding interactions between MEV and immune cell receptors. The tertiary structure of the TLR4-MD2 dimer (TLR4 immune cell receptor) was retrieved from the Protein Data Bank (PDB ID: 4G8A). [23]. Both the immune cell receptor and multi-epitope vaccine structures, in PDB format, were submitted to the HDOCK server for docking analysis. Among the top ten generated models, the model with the most favorable (most negative) docking score was selected for further analysis. The 2D structural interactions between the vaccine protein and TLR4 were subsequently analyzed using Ligplot [49], while 3D visualization and interaction analysis of the docking models were conducted using PyMOL [50].

### 2.12 Basic analysis of the interaction and stability between TLR4 and MEV

The collective motions of the TLR4-MEV docking complex were analyzed using Normal Mode Analysis (NMA) in internal coordinates (torsional space) via the iMODS server (https://imods.iqf.csic.es/) [51]. The dynamic simulation system in iMODS ensured minimal energy configuration, molecular stability, and atomic activity within the TLR4-MEV complex. The PDB file of the TLR4-MEV complex was uploaded to iMODS, and the Coarse Graining (CG) model was selected, with the CA model (representing Cα atoms accounting for whole residue mass) chosen as the atomic model. Basic analysis of the interaction and stability between TLR4 and MEV was subsequently performed, yielding key parameters of the TLR4-MEV complex, including deformability, B-factor, eigenvalues, variance, covariance plots, and elasticity network properties.

### 2.13 Molecular dynamics simulation

Molecular dynamics (MD) simulation is a powerful computational tool used in biomolecular research to study the dynamics, thermodynamics, and interactions of various biological systems at the atomic level. Molecular dynamics (MD) simulations were performed for 100 ns using GROMACS 2025.1 [52]. GROMACS is a widely used free and open-source biomolecular MD simulation software known for its efficiency, accuracy, and extensive simulation options [53]. The TLR4-MEV complex was parameterized with the CHARMM36 force field, solvated in a cubic box under periodic boundary conditions, and hydrated with TIP3P water molecules. Electrostatic interactions were treated using the Particle Mesh Ewald (PME) method with the Verlet cut-off scheme. Van der Waals and Coulombic interactions employed a 1.0 nm cut-off. The system underwent sequential equilibration in the NVT ensemble (310 K) and NPT ensemble (1 atm). All hydrogen-containing bonds were constrained via the LINCS algorithm. Production MD simulations were conducted for 100 ns under NPT conditions with a 2-fs integration time step.

### 2.14 Optimization of MEV codons and in silico cloning

Codon optimization of the MEV sequence was performed using the ExpOptimizer tool (https://www.novopro.cn/tools/codon-optimization.html) with Escherichia coli (E. coli) selected as the expression host, while excluding BamHI and SalI restriction sites. The optimized MEV DNA sequence was subsequently imported into SnapGene 7.1.2, where primers were designed at both ends with the following parameters: length (15–30 bp), GC content (30%−70%), and Tm value (around 60°C) [34]. A BamHI cleavage site (GGATCC) was introduced at the 5’ end of the top strand, and a SalI cleavage site (GTCGAC) was added at the 5’ end of the bottom strand. Polymerase chain reaction (PCR) was simulated in SnapGene 7.1.2 to amplify the optimized DNA sequence, followed by sequence alignment to verify identity between pre- and post-amplification products. For in silico cloning, the amplified vaccine DNA sequence was inserted into the multiple cloning site (MCS) of the pMV261-BCG shuttle plasmid, which is compatible with both E. coli and Mycobacterium tuberculosis expression systems and exhibits adjuvant properties [54]. The cloning process utilized the BamHI and SalI restriction sites. Finally, agarose gel electrophoresis simulations were performed using SnapGene 7.1.2 to analyze the target gene (post-PCR), vector, and recombinant plasmid, with 1% TBE buffer selected for the simulation.

### 2.15 Immunostimulation

C-ImmSim (https://kraken.iac.rm.cnr.it/C-IMMSIM/index.php) [55–57] was employed to simulate the immune response to a multi-epitope vaccine (MEV) targeting brucellosis in sheep. The tool models three key components of the mammalian immune system: the bone marrow, thymus, and lymph nodes, which are essential for B-cell production, T-cell maturation, and immune response coordination, respectively. The simulation parameters were configured as follows: a random seed of 12345 was set to ensure reproducibility, a simulation volume of 50 was selected to represent the scale of the immune system, and a simulation step size of 1050 was used to capture the temporal dynamics of the immune response [32]. Given the high prevalence of brucellosis in sheep in Xinjiang, China, the simulation incorporated specific HLA alleles prevalent in this region: HLA-A\*1101, HLA-A\*0201, HLA-B\*5101, HLA-B\*3501, HLA-DRB1\*0701, and HLA-DRB1\*1501 [23]. These alleles are critical for antigen presentation and were chosen to reflect the genetic background of the target population. The MEV was administered in three doses at time steps 1, 84, and 168, simulating a real-world vaccination schedule. This approach allowed for the observation of the immune system’s response over time, including the activation of B cells, T cells, and the production of antibodies. The primary objective of the simulation was to evaluate the efficacy of the MEV in protecting against brucellosis. By modeling the immune response, we aimed to assess the vaccine’s ability to prime the immune system and potentially reduce the incidence or severity of the disease in the Xinjiang sheep population.

## 3. Result

### 3.1 Selection of target proteins

**Fig 2:**
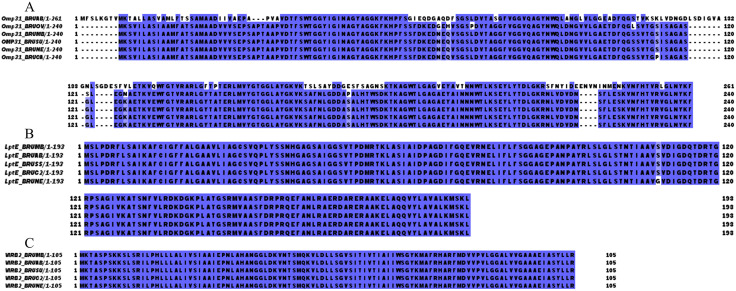
Results of amino acid sequence similarity comparison between Brucella melitensis and other pathogenic Brucella species. **(A)** Sequence comparison results for OMP31. **(B)** Sequence comparison results for LptE. **(C)** Sequence comparison results for VIRB2.

The amino acid sequences of the target proteins were retrieved from the UniProt database, with subcellular localization analysis revealing that several proteins are positioned above the cell membrane. ProtParam analysis provided detailed physicochemical properties for each protein: OMP31 consists of 240 amino acid residues, with a molecular weight of 25323.20 Da, a theoretical pI of 5.22, an instability index (II) of 8.61, and an average hydrophilicity coefficient of −0.091. LptE comprises 193 amino acid residues, with a molecular weight of 20306.23 Da, a theoretical pI of 9.26, an instability index of 20.77, and an average hydrophilicity coefficient of 0.051. VirB2 contains 105 amino acid residues, with a molecular weight of 11107.31 Da, a theoretical pI of 9.99, an instability index of 31.40, and an average hydrophilicity coefficient of 0.728. The instability indices and hydrophilicity coefficients of OMP31, LptE, and VirB2 were below the respective threshold values, indicating that these proteins exhibit favorable stability and hydrophilic properties. Antigenicity analysis using VaxiJen 2.0 yielded scores of 0.6689, 0.6350, and 0.5685 for OMP31, LptE, and VirB2, respectively, all exceeding the threshold of 0.4, confirming their strong antigenic potential. ToxinPred analysis confirmed that all three proteins are non-toxic, while AllergenFP v.1.1 classified them as probable non-allergens. The key properties of these candidate vaccine proteins are summarized in Table 1. Fig 2 visualizes the cross-species sequence identity percentages of OMP31, LptE, and VirB2 in pathogenic *Brucella* species using Jalview software, with detailed sequence identity results provided in S1 Table.

**Table 1:** The key properties of candidate vaccine proteins.

Uniport id	amino acid	Subcellular localization	Antigenicity	Instability index	Allergenicity	Toxicity	Theoretical pi
C0RKW7	OMP31	Cell outer membrane	0.6689	8.61	non-allergen	non-toxicity	5.22
C0RF57	LptE	Cell outer membrane	0.6350	20.77	non-allergen	non-toxicity	9.26
C0RK20	VirB2	intracellular membrane	0.5685	31.40	non-allergen	non-toxicity	9.99

### 3.2 Prediction and excision of signal peptides

**Fig 3:**
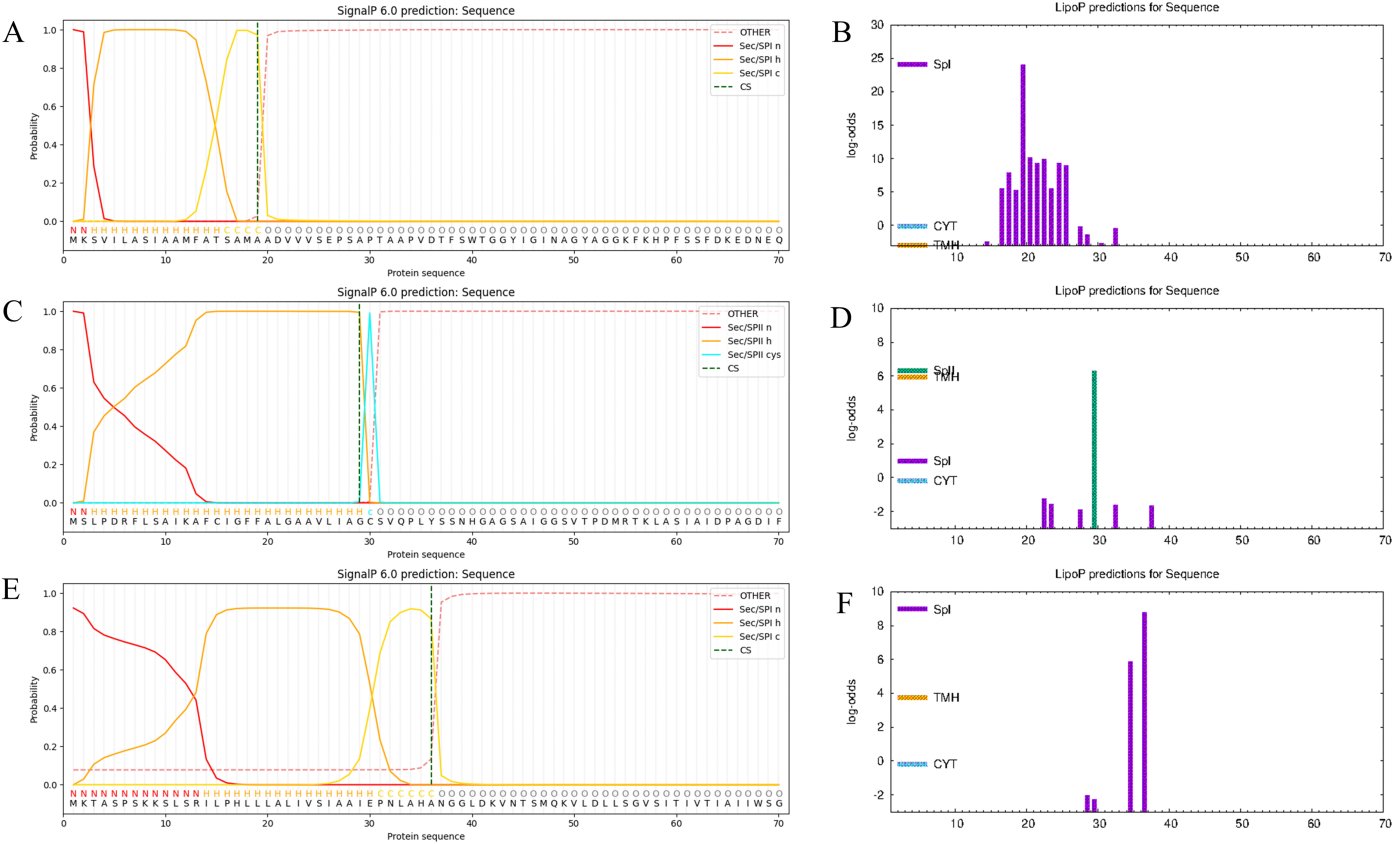
Protein signal peptide prediction results (A-F). **(A)** Predicted OMP31 signal peptide results using SignalP 6.0. **(B)** Predicted OMP31 signal peptide results using LiPOP 1.0. **(C)** Predicted LptE signal peptide results using Signal P 6.0. **(D)** Predicted LptE signal peptide results using LiPOP 1.0. **(E)** Predicted VirB2 signal peptide results using SignalP 6.0. **(F)** Predicted VirB2 signal peptide results using LiPOP 1.0.

SignalP 6.0 and LiPOP 1.0 were used to predict the signal peptides. The signal peptide sequence of OMP31 is MKSVILASIAAMFATSAMAADVVVSEPSAPTAAPVDTFSWTGGYIGINAGYAGGKFKHPFSSFDKE, the signal peptide of LptE is MSLPDRFLSAIKAFCIGFFALGAAVLIAGCSVQPLYSS and the signal peptide of VirB2 is MKTASPSKKSLSRILPHLLLALIVSIAAIEPNLAHAN1(Fig 3). Utimately, we combined the results of these two software programs to remove the signal peptide of three target proteins.

### 3.3 Prediction and selection of T-cell epitopes

Cytotoxic T lymphocyte (CTL) epitopes were predicted using the IEDB database, while helper T lymphocyte (HTL) epitopes were identified using NetMHC-IIpan-4.1. Initially, the top 10 scoring epitopes from each prediction tool were selected for further analysis. These epitopes were subsequently evaluated for allergenicity, antigenicity, instability, and toxicity using AllergenFP v.1.1, VaxiJen2.0, ProtParam, and ToxinPred, respectively. Epitopes that met the top three criteria—high immunogenicity, non-allergenic, non-toxic, and stable—were designated as dominant T-cell epitopes. Based on these stringent criteria, six dominant CTL epitopes and nine dominant HTL epitopes were ultimately selected (Tables 2 and 3). More detailed epitope prediction results are presented in S2–S7 Tables.

**Table 2:** Selected CTLs dominant epitopes of multi-epitope vaccines.

Protein	Epitope types	Alleles	Sequences	Score	Antigenicity	Allergenicity	Toxicity	Instability index	Grand average of hydropathicity (GRAVY)	Theoretical pI
OMP31	CTL epitopes	HLA-A*11:01	GTGGLAYGK	0.7525	0.8821	non-allergen	non-toxicity	−15.13	−0.211	8.59
		HLA-A*02:01	VQAGYNWQL	0.6030	1.8433	non-allergen	non-toxicity	−12.07	−0.367	5.49
		HLA-A*11:01	HTVRVGLNY	0.3516	0.8467	non-allergen	non-toxicity	−8.92	−0.156	8.75
LptE	CTL epitopes	HLA-A*11:01	SVTPDMRTK	0.8525	1.6490	non-allergen	non-toxicity	−17.3	−1.067	8.46
		HLA-A*03:01	SVTPDMRTK	0.6899	1.6490	non-allergen	non-toxicity	−17.3	−1.067	8.46
		HLA-A*11:01	KATSNFVLR	0.2924	1.1164	non-allergen	non-toxicity	13.59	−0.089	11

**Table 3:** Selected HTLs dominant epitopes of multi-epitope vaccines.

Protein	Epitope types	Alleles	Sequences	Score	Antigenicity	Allergenicity	Toxicity	Instability index	Grand average of hydropathicity (GRAVY)	Theoretical pI
OMP31	HTL epitopes	DRB1_0701	VRARLGYTATERLMV	0.8258	0.7145	non-allergen	non-toxicity	10.85	0.093	10.74
		DRB1_0701	RARLGYTATERLMVY	0.8132	0.5659	non-allergen	non-toxicity	5.83	−0.273	9.98
		DRB1_0701	GAEYAINNNWTLKSE	0.6005	1.3109	non-allergen	non-toxicity	9.71	−0.907	4.53
LptE	HTL epitopes	DRB1_0301	IGGSVTPDMRTKLAS	0.9375	0.7282	non-allergen	non-toxicity	−3.81	−0.073	8.75
		DRB1_0301	GGSVTPDMRTKLASI	0.9374	0.8665	non-allergen	non-toxicity	−3.81	−0.073	8.75
		DRB1_0701	PSAGIVKATSNFVLR	0.8631	0.4667	non-allergen	non-toxicity	11.75	0.46	11.01
VirB2	HTL epitopes	DRB1_0701	LGGALVVGAAAEIAS	0.2689	0.5069	non-allergen	non-toxicity	−3.81	1.6	4.0
		DRB1_0701	GGALVVGAAAEIASY	0.2613	0.4919	non-allergen	non-toxicity	−3.81	1.26	4.0
		DRB1_0701	VLGGALVVGAAAEIA	0.1642	0.5320	non-allergen	non-toxicity	11.75	1.933	4.0

### 3.4 Prediction and selection of B-cell epitopes

**Fig 4:**
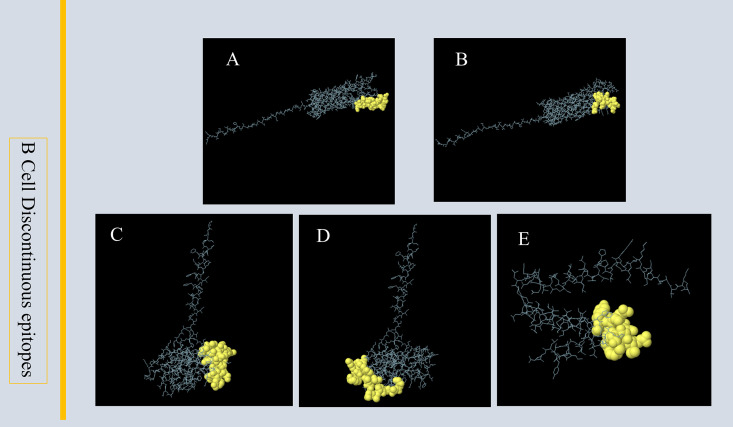
B-cell conformational epitopes (A-E). **(A-B)** B-cell conformational epitope residues of OMP31. **(C-D)** B-cell conformational epitope residues of LptE. **(E)** B-cell conformational epitope residues of VirB2.

Linear B-cell epitopes were predicted and analyzed using the IEDB and ABCpred servers, leading to the selection of seven dominant linear epitopes. Additionally, conformational B-cell epitopes were identified using the Ellipro online server from IEDB, resulting in five dominant conformational epitopes (Fig 4). All selected epitopes exhibited high scores, strong antigenicity, stability, and were confirmed to be non-allergenic and non-toxic (Tables 4 and 5). These characteristics underscore their potential as effective components for vaccine design. The original predictions are presented in S8–S13 Tables.

**Table 4:** Selected LBEs dominant epitopes of multi-epitope vaccines.

Protein	Start position	LBEs epitopes	Score	Antigenicity	Allergenicity	Toxicity	Instability index	Grand average of hydropathicity (GRAVY)	Theoretical PI
OMP31	108	LHTWSDKTKAGWTLGA	0.90	0.8622	non-allergen	non-toxicity	−17.03	−0.55	8.6
	118	GWTLGAGAEYAINNNW	0.89	1.0419	non-allergen	non-toxicity	5.4	−0.331	4
	46	TGSISAGASGLEGKAE	0.88	1.6736	non-allergen	non-toxicity	−6.54	−0.119	4.53
LptE	5	GSAIGGSVTPDMRTKL	0.90	0.9958	non-allergen	non-toxicity	−2.95	−0.094	8.75
	73	DIGDQTDRTGRPSAGI	0.87	1.237	non-allergen	non-toxicity	18.12	−1.075	4.43
	64	TNTIAAVSVDIGDQTD	0.81	0.5291	non-allergen	non-toxicity	−14.72	0.013	3.42
VirB2	1	GGLDKVNTSMQKVLDL	0.60	0.5398	non-allergen	non-toxicity	−8.85	−0.15	5.96

**Table 5:** Selected CBEs dominant epitopes of multi-epitope vaccines.

Protein	CBEs epitopes	Residues	Score	Antigenicity	Allergenicity	Toxicity	Instability index	Grand average of hydropathicity (GRAVY)	Theoretical PI
OMP31	KPFSSFDKEDNEQVSGSLDNSFE	K57, P59, F60, S61, S62, F63, D64, K65, E66, D67, N68, E69, Q70, V71, S72, G73, S74, L75, D76, N220, S221, F222, E224	0.785	0.5306	non-allergen	non-toxicity	38.18	−1.257	4.02
	EANLGDDASALHTW	E127, A164, N166, L167, G168, D169, D170, A171, S172, A173, L174, H175, T176, W177	0.6442	0.6442	non-allergen	non-toxicity	−6.84	−0.5	4.02
LptE	AAVSVDIGDQTDTGRPSADRPQEFANLR	A106, A107, V108, S109, V110, D111, I112, G113, D114, Q115, T116, D117, T119, G120, R121, P122, S123, A124, D155, R156, P157, Q159, E160, F161, A162, N163, L164, R165	0.787	0.4913	non-allergen	non-toxicity	31.42	−0.779	4.36
	SGGAGEPANPAYRDKDGKPL	S60, G84, G85, A86, G87, E88, P89, A90, N91, P92, A93, Y94, R136, D137, K138, D139, G140, K141, P142, L143.	0.683	1.1114	non-allergen	non-toxicity	14.39	−1.28	5.84
VirB2	GYKMAFRARFMDVV	G70, Y71, K72, M73, A74, F75, R76, A78, R79, F80, M81, D82, V83, V84	0.699	1.0409	non-allergen	non-toxicity	35.32	0.236	9.99

### 3.5 Molecular docking analysis of CTL and HTL epitopes with HLA alleles

**Fig 5:**
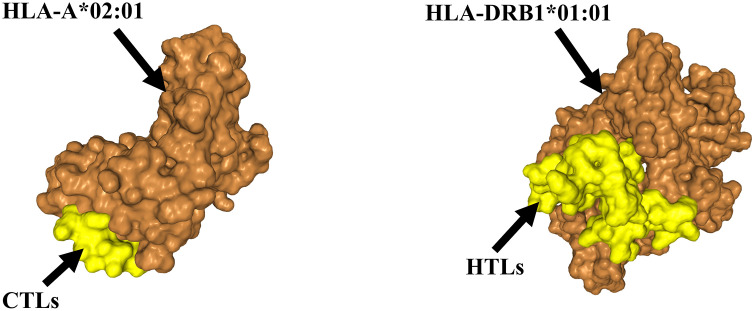
The docked results of T cell epitopes with HLA (A-B). **(A)** Molecular docking result of CTL epitopes to HLA-A*02:01. **(B)** Molecular docking result of HTL epitopes to HLA-DRB1*01:01.

Molecular docking was performed to investigate the interactions between cytotoxic T lymphocyte (CTL) and helper T lymphocyte (HTL) epitopes and their corresponding antigen-presenting cell (APC) HLA alleles. Among the top ten models, the one with the most negative docking score was selected for detailed analysis. For CTL epitopes interacting with HLA-A*02:01, the docking score was −205.76, with a confidence score of 0.7531 and a ligand RMSD of 23.50 Å. For HTL epitopes interacting with HLA-DRB1*01:01, the docking score was −269.66, with a confidence score of 0.9163 and a ligand RMSD of 49.70 Å. The docking results demonstrated strong binding affinity between HLA molecules and T-cell epitopes (Fig 5), indicating that these epitopes can be efficiently presented to T-cells for immune recognition.

### 3.6 Construction of the multi-epitope vaccine

**Fig 6:**
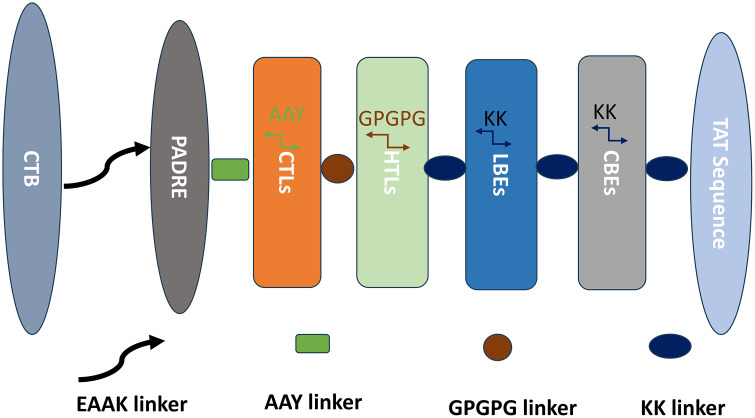
Multi-epitope vaccine construction diagram.

The multi-epitope vaccine was designed by linking selected dominant antigenic epitopes of T-cells and B-cells with adjuvants using appropriate linkers. The final vaccine construct comprised six cytotoxic T lymphocyte (CTL) epitopes, nine helper T lymphocyte (HTL) epitopes, six linear B-cell epitopes (LBE), five conformational B-cell epitopes (CBE), cholera toxin B subunit (CTB), pan HLA-DR epitope (PADRE), and the TAT-sequence. The schematic representation of the constructed vaccine is illustrated in Fig 6.

### 3.7 Physicochemical and functional properties of the multi-epitope vaccine

The physicochemical and functional properties of the constructed multi-epitope vaccine (MEV) were analyzed using computational tools. ProtParam analysis revealed that the MEV has a molecular formula of C2849H4565N809O863S16, a molecular weight of 64472.45 Da, a theoretical pI of 9.70, and consists of 621 amino acids. The instability index (II) of 12.43, which is below the stability threshold of 40, indicates that the MEV is a stable protein. Furthermore, the GRAVY value of −0.319 confirms its hydrophilic nature. The aliphatic index of MEV is 73.08. Antigenicity prediction using VaxiJen2.0 yielded a score of 0.8157, significantly exceeding the threshold of 0.4, demonstrating strong antigenic potential. AllergenFP v.1.1 classified the MEV as a probable non-allergen, confirming its non-sensitizing properties. Solubility analysis using the SOLpro server resulted in a score of 0.8703, surpassing the threshold of 0.5, indicating high solubility. Collectively, these analyses confirm that the constructed MEV is stable, hydrophilic, antigenic, non-allergenic, and soluble, making it suitable for further investigation. The detailed physicochemical and functional parameters of the MEV are summarized in Table 6.

**Table 6:** Properties of designed multi-epitope vaccines.

No	Physiochemical profifiling	Measurement	Indication
1	Number of amino acids	621	Appropriate
2	Formula	C_2849_H_4565_N_809_O_863_S_16_	—
3	Molecular weight	64472.4Da	Appropriate
4	Theoretical pi	9.70	—
5	Total number of atoms	9102	—
6	Total number of negatively charged residues (Asp + Glu)	53	—
7	Total number of positively charged residues (Arg + Lys)	82	—
8	Instability index (II)	12.43	Stable
9	Aliphatic index	73.08	—
10	Grand average of hydropathicity (GRAVY)	−0.319	Hydrophilic
11	Antigenicity	0.8157	Antigenic
12	Allergenicity	Non-Allergen	—
13	Solubility	0.870	Soluable

### 3.8 Secondary and tertiary structure prediction of MEV

**Fig 7:**
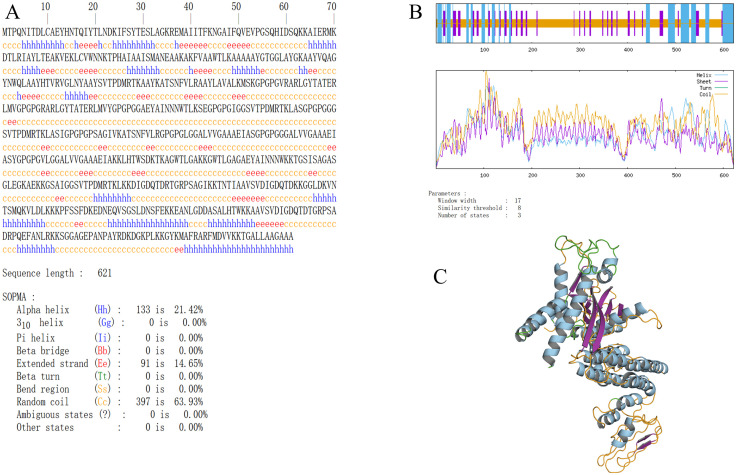
Prediction of secondary and tertiary structures of multi-epitope vaccines(A-C). **(A-B)** Prediction results of MEV secondary structure. **(C)** pymol visualization of Robetta prediction of MEV tertiary structure results.

Secondary structure prediction of the multi-epitope vaccine (MEV) using SOPMA revealed a composition of 21.42% α-helices, 63.93% random coils, and 14.65% extended strands (Fig 7A and 7B). The tertiary structure was predicted using the Robetta platform (RoseTTAFold), with Model 1 selected as the optimal structure and visualized in PyMOL (Fig 7C). Comparative analysis of the secondary and tertiary structures demonstrated strong concordance, validating the accuracy of the structural prediction and confirming the reliability of the vaccine protein’s modeled conformation.

### 3.9 Refinement and validation of the tertiary structure of MEV

**Fig 8:**
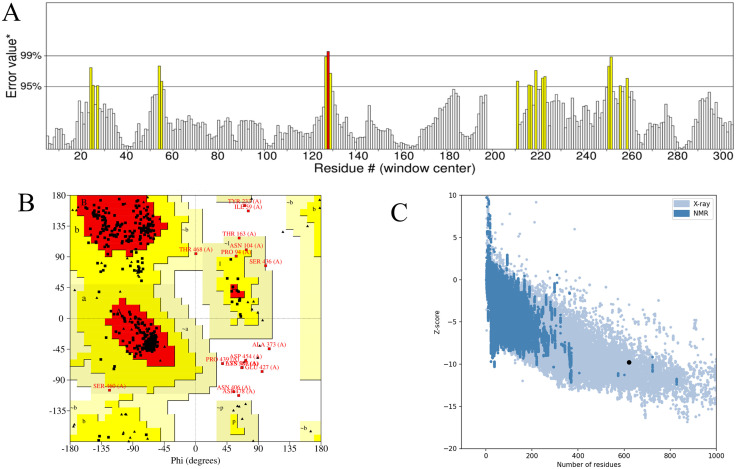
Prediction and refinement of tertiary structures of multi-epitope vaccines(A-C). **(A)** Quality factor plot for the improved vaccine protein. **(B)** Ramachandran Plot of MEV tertiary structure after refinement. **(C)** Z-value plot for the improved vaccine protein.

The tertiary structure of the multi-epitope vaccine (MEV) was refined using the GalaxyRefine server, which generated five refined models (S14 Table). Model 2 was selected as the optimal refined structure based on its superior parameters: Rama favored (94.2%), poor rotamers (0.7%), MolProbity score (1.976), clash score (11.7), RMSD (0.317), and GDT-HA score (0.9847). ERRAT analysis further validated the quality of the refined structure, yielding a quality factor of 92.70% (Fig 8A). The Ramachandran plot analysis revealed that 90.3% of the amino acids were in favored regions, 6.9% in additional allowed regions, 0.8% in generously allowed regions, and 2.0% in disallowed regions (Fig 8B). Additionally, ProSA-web analysis of Model 1 resulted in a Z-value of −9.78 (Fig 8C), confirming the structural reliability and quality of the refined MEV model. These results collectively demonstrate the high accuracy and stability of the refined tertiary structure.

### 3.10 Disulfide bond engineering of construct and evaluation

**Fig 9:**
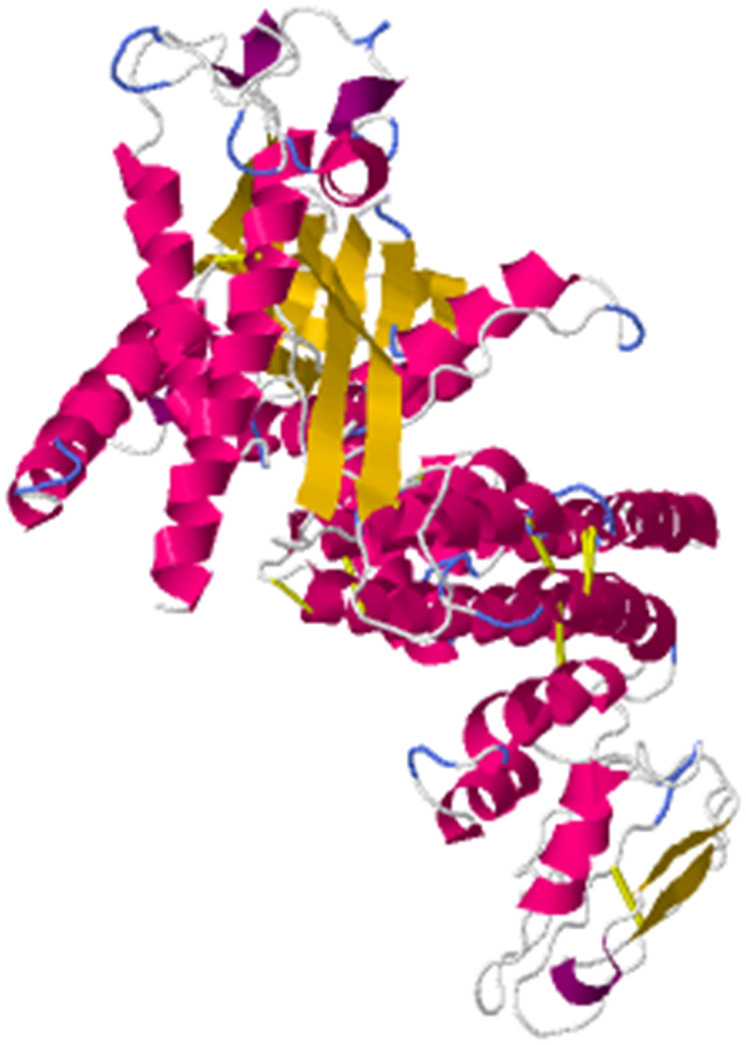
Disulfide bond engineering results for vaccines. Vaccine construct after disulfide bond engineering. The thin yellow lines in the figure indicate constructed disulfide bonds.

Analysis using the Disulfide by Design server identified 68 potential residue pairs for disulfide bond formation in the vaccine protein. After applying stringent criteria based on χ3 angles and calculated energy fractions, 11 optimal disulfide bond pairs were selected (S15 Table): ILE6-LYS85, TYR28-TYR77, ALA114-ALA139, ALA158-ALA170, THR163-MET166, ARG221-VAL232, ASN309-ALA329, ALA328-ALA347, ILE331-ALA369, ALA332-ALA373, and SER546-ARG601. As demonstrated in Fig 9, the structure of the vaccine with the addition of a disulphide bond is illustrated and Post-engineering structural validation was performed using ProSA-web and ERRAT. The Z-value and quality factor after disulfide bond incorporation were −9.51 and 92.83%, respectively (S1 and S2 Figs), compared to −9.78 and 92.70% prior to engineering. This comparison demonstrates that the introduction of disulfide bonds enhanced the structural quality and stability of the vaccine model, further validating the effectiveness of the engineering approach.

### 3.11 Protein-protein docking

**Fig 10:**
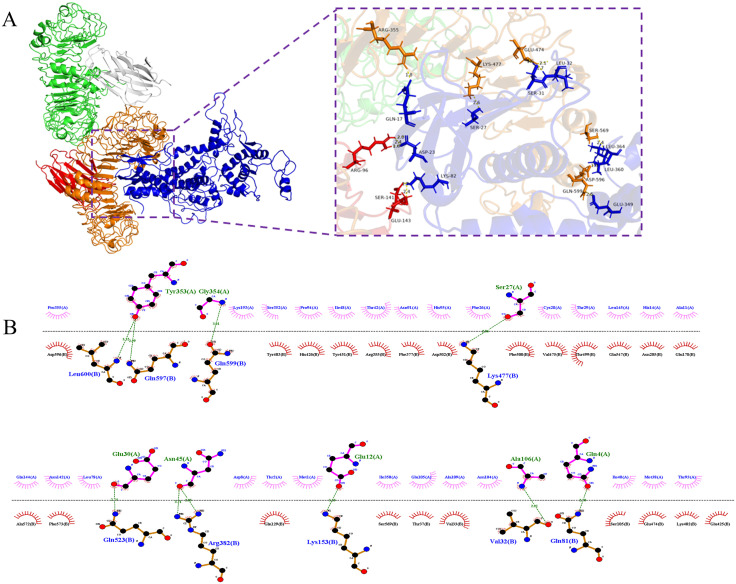
The docking results and interaction of TLR4-MEV (A-B). **(A)** The docking of the TLR4 -MEV complex using Pymol to demonstrate. **(B)** Result of the interaction of TLR4-MEV complex and its 2D image using Ligplot + v.2.2.9. Hydrogen bonds are represented by dotted green lines, and red semicircles indicate residues involved in hydrophobic interactions.

Molecular docking between the multi-epitope vaccine (MEV) and the immune cell receptor TLR4 was performed using the HDOCK server. Among the top ten models, Model 1, characterized by the most negative docking score, was selected as the optimal model for further analysis. The docking results for Model 1 revealed a docking score of −311.85, an RMSD of 91.81 Å for the ligand, and a confidence level of 0.9622. The docking structure and 3D interactions were visualized using PyMOL, demonstrating the formation of 16 hydrogen bonds between TLR4 and MEV (Fig 10A). Additionally, the 2D interaction interface was analyzed using LigPlot+ v.2.2.9, highlighting salt bridges (red dashed lines) and hydrogen bonds (green dashed lines) at the binding site (Fig 10B). These results indicate a strong and stable interaction between TLR4 and MEV, suggesting the vaccine’s potential to effectively engage the immune receptor and elicit a robust immune response.

### 3.12 Basic analysis result of the interaction and stability between TLR4 and MEV

**Fig 11:**
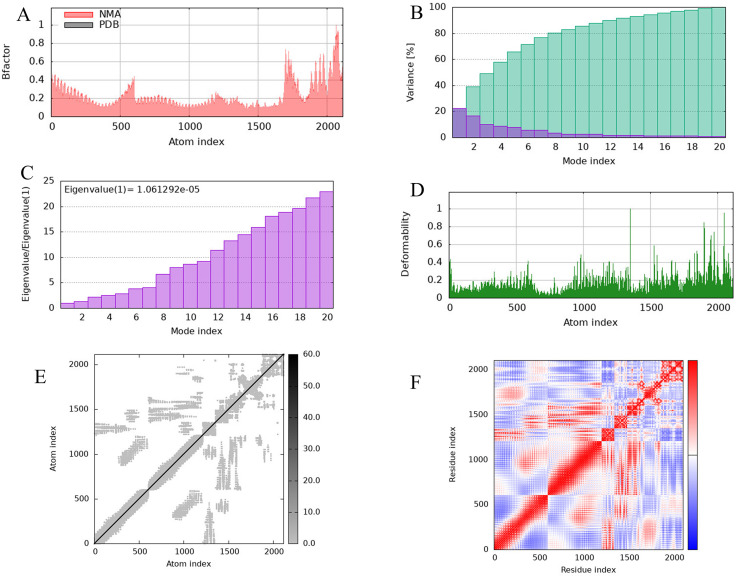
Basic analysis result of the interaction and stability between TLR4 and MEV. **(A)** B-factor and NMA graph. **(B)** The plot variance associated to the each modes. **(C)** Eigenvalues plot. **(D)** The complex residue deformability graph. **(E)** The elastic network models plot. **(F)** Covariance matrix graph.

Basic analysis of the interaction and stability between TLR4 and MEV complex were conducted using the IMODS server to evaluate stiffness, mobility, and deformability of the complex residues. Normal mode analysis (NMA) was employed to assess the dynamic behavior of the complex. The B-factor plot (Fig 11A) illustrates the correlation between NMA mobility and the corresponding PDB field, where the B-factor is derived by multiplying the NMA mobility by (8π²). The variance plot (Fig 11B) demonstrates the variance associated with each normal mode, which inversely correlates with the eigenvalue, showing a gradual decrease in individual variance across modes. The calculated eigenvalue of the TLR4-MEVcomplex is 1.061292e-05, indicating the energy required for structural deformation and reflecting a gradual increase in continuum mode (Fig 11C). Residue mobility analysis (Fig 11D) highlights the deformability of the complex at each residue. The elastic network model (Fig 11E) depicts atom pairs connected by springs, with darker gray dots representing stiffer springs. The covariance matrix (Fig 11F) illustrates the coupling between residue pairs, where red indicates correlated motion, white uncorrelated motion, and blue anticorrelated motion. These analyses collectively provide insights into the dynamic stability and flexibility of the TLR4-MEV complex, supporting its potential for effective immune receptor engagement.

### 3.13 Molecular dynamics simulation results

**Fig 12:**
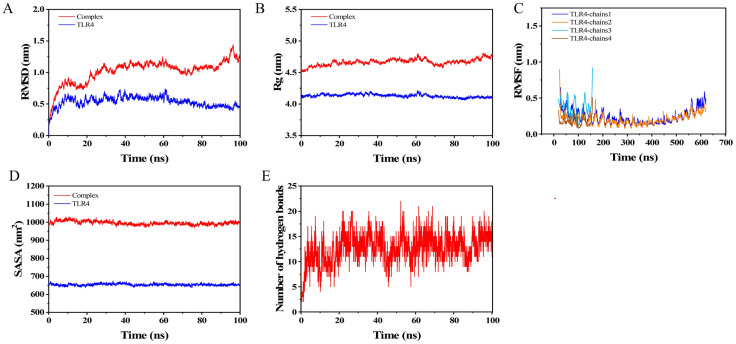
Molecular dynamics simulation results. **(A)** RMSD plots of TLR4 and the complex. **(B)** Rg plots of TLR4 and the complex. **(C)** RMSF plots of the four chains of the TLR4 protein bound to MEV. **(D)** SASA plots of TLR4 and the complex. **(E)** Hydrogen bond plots of the complex.

Molecular dynamics (MD) simulations were performed for TLR4 and the complex using GROMACS 2025.1. Root mean square deviation (RMSD) is a reliable indicator for assessing conformational stability of proteins and ligands. RMSD evaluated system equilibration. As shown in Fig 12A, both TLR4 and complex(TLR4-MEV) groups reached equilibrium at ~40 ns. The complex system fluctuated around 1.0 nm with small RMSD values and minor fluctuations, indicating system stabilization. Radius of gyration (Rg) reveals structural stability before/after ligand binding, complementing RMSD. Fig 12B shows both groups remained stable with minimal differences throughout 0–100 ns simulation. Root mean square fluctuation (RMSF) measures positional variation per residue (Fig 12C). The TLR4 chains showed small fluctuations, indicating stability. Solvent-accessible surface area (SASA) estimates amino acid solvent exposure (Fig 12D). SASA values remained relatively constant, suggesting system stability. Fig 12E shows hydrogen bonds (0–21) between protein and ligand. Most simulations maintained 10–15 bonds.

### 3.14 Codon optimization and In Silico cloning of the MEV protein

**Fig 13:**
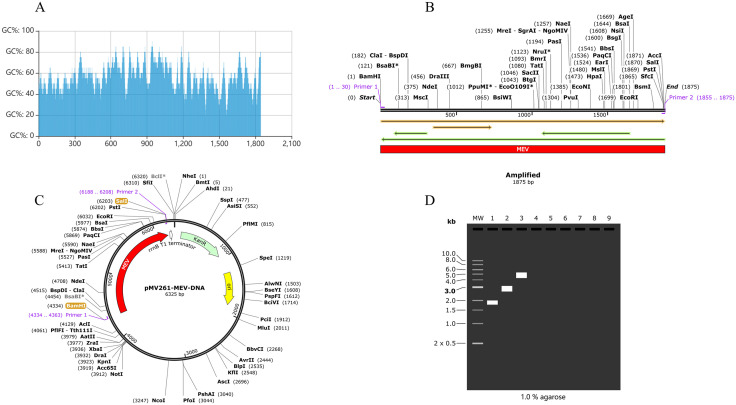
The result of Optimization of MEV codons and in silico cloning (A-D). **(A)** The GC content after codon optimization is 53.73%. **(B)** MEV DNA sequence of after PCR. **(C)** The red region is the amplified target gene sequence of MEV inserted into Escherichia coli-BCG shuttle plasmid pMV261. **(D)** Simulated agarose gel electrophoresis results. “1” represents MEV after PCR, “2” represents shuttle plasmid pMV261, “3” represents pMV261-MEV-DNA recombinant plasmid.

To enhance the expression of the vaccine protein, the MEV amino acid sequence was optimized using the ExpOptimizer tool. The optimized sequence was back-translated into a 1863-base DNA sequence, achieving a codon adaptation index (CAI) of 0.81 and a GC content of 56.20% (Fig 13A). Primers for polymerase chain reaction (PCR) amplification were designed using SnapGene 7.1.2. Primer 1 (5′- ATGACCCCGCAAAACATTACCGAC-3′) on the top strand had a length of 24 bp, a Tm value of 62°C, and a GC content of 50%. Primer 2 (5′-TGCAGCCGCGCCAGC-3′) on the bottom strand had a length of 15 bp, a Tm value of 64°C, and a GC content of 80%. The MEV DNA sequence was successfully amplified via PCR (Fig 13B), with no specific cleavage sites identified in the target protein sequence. The amplified MEV gene sequence was subsequently inserted into the multiple cloning site (MCS) of the Escherichia coli-BCG shuttle plasmid pMV261, completing the in silico cloning process (Fig 13C). Simulated agarose gel electrophoresis confirmed the expected sizes: the MEV-PCR sequence was 1875 bp, the pMV261 plasmid sequence was 4488 bp, and the recombinant pMV261-DNA-MEV plasmid sequence was 6325 bp (Fig 13D). These results align with the anticipated outcomes, validating the successful optimization and cloning of the MEV protein.

### 3.15 Immunostimulation

**Fig 14:**
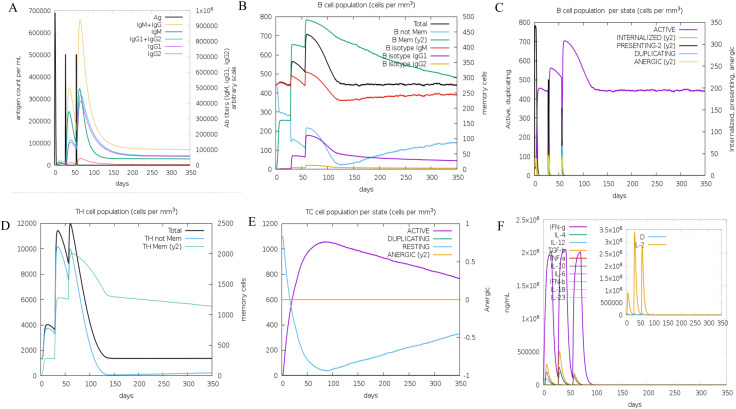
Immunostimulation simulation results(A-F). **(A)** Number of immunoglobulins produced after 3 injections. **(B)** The B cell population after 3 injections. **(C)** The B cell population per state after 3 injections. **(D)** The number of helper T cells after 3 injections. **(E)** The cytotoxic T cells population per state after 3 injections. **(F)** Number of cytokines and interleukins after 3 injections.

The immune response to three doses of the vaccine was simulated using C-ImmSim. Fig 14A illustrates that antibody concentrations increased rapidly after each injection, peaking after the third dose and subsequently stabilizing at a lower level. B cells, a critical component of humoral immunity, exhibited a similar trend, with their numbers rising progressively and peaking after the third injection (Fig 14B). Fig 14C shows a rapid increase in activated B cells after three doses of the vaccine, reaching a peak of 700 cells/mm^3^, followed by a gradual decline to 450 cells/mm^3^ and continued stabilisation until day 350, also indicating that the vaccine successfully activated B cells and produced a sustained immune response. Helper T (TH) cells, essential for immune enhancement, also increased in number with each vaccine dose, reaching a peak after the third injection before stabilizing (Fig 14D). Fig 14E shows a rapid increase in activated TC cells to 1050 cells/mm^3^ after vaccination, followed by a slow decrease to 780 cells/mm^3^ on day 350. Cytokine and interleukin production surged after three vaccine doses. Notably, IFN-γ reached a peak of 2 × 10^6 ng/ml, while TGF-β, IL-10, IL-12, and IL-2 also showed significant elevations (Fig 14F). These results collectively demonstrate a robust and sustained immune response to the vaccine, highlighting its potential efficacy. The results of immune simulation of other immune cells (non-memory cytotoxic T cells, natural killer cells, dendritic cells, activated macrophages, activated eosinophils) are shown in the S3 Fig.

## 4. Discussion

Brucella, a Gram-negative intracellular pathogen, remains a significant global health concern, causing zoonotic brucellosis in humans and animals with substantial economic impacts [58]. Current estimates indicate approximately 500,000 new human cases annually [59]. Despite its global burden, no licensed human vaccine against Brucella exists, underscoring the urgent need for developing safe and effective vaccination strategies [60]. The emergence of bioinformatics and reverse vaccinology approaches has revolutionized vaccine development, particularly through the design of recombinant multi-epitope vaccines [61]. In this study, we identified three promising vaccine candidates: the outer membrane protein OMP31, lipopolysaccharide transporter protein LptE, and type IV secretion system protein VirB2. OMP31, a well-characterized virulence factor, has demonstrated protective efficacy against Brucella infection in sheep models and exhibits strong immunogenic properties [62,63]. Lipopolysaccharides (LPS), essential components of Gram-negative bacteria, are critical for bacterial survival under adverse conditions, with their membrane integration mediated by the LptD-LptE translocator system [64,65]. The type IV secretion system (T4SS), particularly its surface-exposed components VirB2 and VirB5, plays a pivotal role in effector protein delivery and intracellular survival of Brucella [66], while remaining accessible to antibody-mediated immunity [67]. Our bioinformatic analysis revealed that all three target proteins exceeded the antigenicity threshold of 0.4, demonstrating favorable immunogenic potential. Furthermore, these proteins exhibited optimal characteristics regarding stability, allergenicity, and toxicity profiles. Analysis of the similarity of these proteins among Brucella pathogenic species indicates that they are highly similar among Brucella pathogens and can meet the requirements for protection against multiple pathogenic species. This study targets multiple critical pathogenic processes in Brucella infection—including adhesion, immune evasion, and intracellular survival—by selecting highly conserved proteins and strategically balancing both antibody and cellular immune responses [6]. This integrated approach aims to enhance vaccine efficacy and protection, contrasting with prior research primarily focused on individual processes [68,69].

Signal peptides, typically comprising 20−25 amino acid residues, play a crucial role in protein secretion across prokaryotic and eukaryotic systems, where they facilitate the synthesis of terminally extended precursors [70]. In this study, we employed SignalP6.0 and LiPOP1.0 to predict and subsequently remove the signal peptides from our three vaccine candidate proteins, as these sequences are non-essential for multi-epitope vaccine construction. The rapid advancement of immunoinformatics, powered by machine learning and comprehensive databases, has revolutionized vaccine design, enabling efficient screening and characterization of highly immunogenic peptides and epitopes [71]. The adaptive immune system, particularly through lymphocyte-mediated responses, provides specific and memory-driven protection against pathogens [72]. To harness this mechanism, we utilized IEDB and NetMHCIIpan-4.1 for cytotoxic T lymphocyte (CTL) and helper T lymphocyte (HTL) epitope prediction, while B-cell epitopes were identified through IEDB, ABCpred, and Ellipro analysis. Our comprehensive screening yielded 6 CTL epitopes, 9 HTL epitopes, 7 linear B-cell epitopes, and 5 conformational B-cell epitopes, all demonstrating optimal immunogenicity, stability, and safety profiles. The integration of immunoinformatics has significantly enhanced our understanding of MHC-mediated antigen presentation, a cornerstone of adaptive immunity [73]. Our analysis confirmed strong MHC binding affinity for both HTL and CTL epitopes. Despite the rich HLA polymorphism, core high-frequency alleles dominate the immune response pattern of the population through linkage haplotypes. This design covers 68.75% of individuals and more than 80% of response potential, and can provide vaccine protection for most people [22]. To optimize vaccine efficacy, we strategically incorporated CTB and PADRE adjuvants at the N-terminus, connected via appropriate linkers, to enhance antigenicity and immunogenicity. Recognizing Brucella’s intracellular survival strategy [74], we incorporated a TAT sequence at the C-terminus to facilitate cellular penetration [36], completing the construction of our multi-epitope vaccine candidate.

Physicochemical characterization of the multi-epitope vaccine revealed favorable properties, with an instability index of 12.43 and a hydrophilicity score of −0.319, indicating structural stability and hydrophilic nature. The molecular weight of 64.5 kDa, well below the 110 kDa threshold [75], satisfies essential vaccine design criteria. Comprehensive analysis demonstrated the vaccine’s non-allergenic nature and strong antigenicity, supported by a solubility prediction score of 0.8703, significantly exceeding the 0.5 threshold. These results confirm the successful construction of a vaccine meeting all design specifications.Structural analysis was performed using SOMPA for secondary structure prediction and Robetta for tertiary structure modeling. Subsequent refinement through Galaxy Refine yielded an optimized structure, validated by Ramachandran Plot, Prosweb, and Z-value analyses. To enhance structural stability, we engineered disulfide bonds using the Disulfide by Design server, selecting 11 optimal pairs with energies below 2.0 kcal/mol [28]. Comparative ERRAT and ProSA-web evaluations demonstrated significant improvement in structural quality following disulfide bond incorporation. The vaccine’s immunogenic potential was further validated through molecular docking studies using the HDOCK server. Although PRRs such as TLR2 and TLR9 participate in Brucella recognition, TLR4 is the preferred target due to its important role in Brucella and its synergistic effect with BCG [76,77]. NMA analysis results confirmed structural stability, with low deformation propensity, B-factor values, and eigenvalues, indicating a robust and stable vaccine-receptor complex. Molecular dynamics simulations showed that stable RMSD (~1.0 nm) and unchanged Rg confirm the structural rigidity of the complex. Low RMSF and constant SASA indicate no excessive fluctuation in epitope regions or exposure of the hydrophobic core. Sustained hydrogen bonding (10–15 bonds) and defined binding residues reveal strong specific interactions between the vaccine and TLR4. These results demonstrate good structural stability and consistent binding interactions for TLR4-MEV.

Codon optimization was performed using ExpOptimizer, yielding a 1863 bp DNA sequence with a GC content of 53.73% and a codon adaptation index (CAI) of 0.79. Enzymatic cleavage sites, BamHI and SalI, were incorporated into the top and bottom strands, respectively, to facilitate cloning and amplification. The optimized sequence was subsequently inserted into the pMV261-BCG shuttle vector, leveraging BCG as an adjuvant due to its safety, stability, cost-effectiveness, and capacity to induce non-specific cross-protection [78,79]. BCG live carriers enable lymph node-targeted delivery, while GPGPG flexible linkers enhance epitope accessibility [80]. Molecular dynamics simulations demonstrate that they can stably bind to TLR4, theoretically enhancing vaccine efficacy. Immune response simulation using the C-ImmSim server demonstrated robust immunogenicity, with immunoglobulin levels peaking rapidly after three doses. The vaccine effectively induced immune memory, a critical feature of antigen exposure, particularly through the activation of B cells and T helper (TH) cells [81]. Post-vaccination, B cells and TH cells exhibited an initial surge followed by stabilization, indicative of sustained immune activation. Notably, B cells differentiated into plasma cells, leading to significant antibody production. The vaccine also elicited a strong cytokine response, with elevated levels of IFN-γ, TGF-β, IL-10, IL-12, and IL-2, underscoring its ability to trigger a comprehensive immune response. In summary, the multi-epitope vaccine demonstrated excellent physicochemical properties, strong binding affinity to immune cell receptors, and the capacity to elicit a robust host immune response. These findings highlight its potential as a promising candidate for Brucella vaccine development and provide a solid theoretical foundation for further preclinical and clinical studies.

## 5. Conclusion

This study computationally designed a novel BCG multi-epitope vaccine targeting key immunogenic proteins for Brucella. The engineered construct demonstrated good antigenicity, stability, and solubility, and showed strong binding affinity with immune receptors in vitro simulations. The construct was successfully cloned into the BCG shuttle vector in software, generating a recombinant vaccine candidate. Immunological simulation predicts that it can induce robust and sustained immune activation. These results lay a promising foundation for the development of a Brucella vaccine.

## 6. Limitation

This study relies exclusively on in silico predictions, which carry inherent limitations: (1) experimental validation of immunogenicity and protective efficacy is required; (2) simplified immune modeling cannot fully replicate human immune complexity; (3) translational uncertainty exists regarding the in vivo behavior of the recombinant BCG construct. Future research must therefore prioritize laboratory validation to advance this vaccine concept.

## Supporting information

S1 Table Clustal Omega multiple sequence alignment results for OMP31、LptE and VIRB2. (DOCX)

S2 Table MHC-I binding prediction results of OMP31 (IEDB). (DOCX)

S3 Table MHC-I binding prediction results of LptE (IEDB). (DOCX)

S4 Table MHC-I binding prediction results of VirB2 (IEDB). (DOCX)

S5 Table MHC- II binding prediction results of OMP31(NetMHC-IIpan-4.1). (DOCX)

S6 Table MHC- II binding prediction results of LptE(NetMHC-IIpan-4.1). (DOCX)

S7 Table MHC-Ⅱ binding prediction results of VirB2(NetMHC-IIpan-4.1). (DOCX)

S8 Table LBEs results of OMP31(ABCpred and IEDB). (DOCX)

S9 Table LBEs results of LptE (ABCpred and IEDB). (DOCX)

S10 Table LBEs results of VirB2 (ABCpred and IEDB). (DOCX)

S11 Table CBEs results of OMP31 (IEDB). (DOCX)

S12 Table CBEs results of LptE (IEDB). (DOCX)

S13 Table CBEs results of VirB2 (IEDB). (DOCX)

S14 Table Tertiary structure results of multi-epitope vaccines optimised by GalaxyRefine. (DOCX)

S15 Table Screening of disulfide bonds by using the Disulfide by Design server. (DOCX)

S1 Fig The plot of Z-value after disulfide bond incorporation. (TIF)

S2 Fig The plot of quality factor after disulfide bond incorporation. (TIF)

S3 Fig Immune simulation results of other immune cells. (TIF)
