# Correction: Design of a multi-epitope recombinant BCG vaccine targeting Brucella OMP31, LptE and VirB2 in immunoinformatics approaches

**DOI:** 10.1371/journal.pone.0342000

**Published:** 2026-01-29

**Authors:** The PLOS One Staff

There are errors in the author affiliations. The correct affiliations are as follows:

Chuang Li^1^, Yuejie Zhu^2^, Xingxing Qi^3^, Zhenglong Chai^1^, Jiarui Luo^1^, Kaiyu Shang^1^, Tingting Tian^1^, Huidong Shi^1^, Mingzhe Li^4^, Ruixue Xu^4^, Fuling Pu^4^, Junyu Kuang^4^, Fengbo Zhang ^1,5^

1 State Key Laboratory of Pathogenesis, Prevention and Treatment of High Incidence Diseases in Central Asia, The First Affiliated Hospital of Xinjiang Medical University, Urumqi, China, 2 Reproductive Medicine Center, The First Affiliated Hospital of Xinjiang Medical University, Urumqi, China, 3 Post-doctoral Research station of the Clinical Medicine, The First Affiliated Hospital of Xinjiang Medical University, Urumqi, China, 4 Department of Medicine, The First Clinical Medical College of Xinjiang Medical University, Urumqi, China, 5 Department of Clinical Laboratory, The First Affiliated Hospital of Xinjiang Medical University, Xinjiang, China.

The publisher apologizes for the error.
