# Supplementary material for: Design of a multi-epitope recombinant BCG vaccine targeting Brucella OMP31, LptE and VirB2 in immunoinformatics approaches
Source: PLoS One. 2025 Nov 6;20(11):e0334843. doi: 10.1371/journal.pone.0334843 (PMC12591482; doi:10.1371/journal.pone.0334843)
Supplement: S1 Table — (DOCX) [file pone.0334843.s001.docx]

**Clustal Omega multiple sequence alignment results for core pathogenic Brucella species：OMP31、LptE and VIRB2**

1. **OMP31 Results:**

| **Serial number** | **Species** | **UniProt ID-** | **UniProt ID -Species** | **Sequence identity (%)** |
| --- | --- | --- | --- | --- |
| 1 | **B. melitensis** | C0RKW7 | C0RKW7_BRUMB | **100.00** |
| 2 | B. abortus | F8WJS1 | F8WJS1_BRUAB | **67.51** |
| 3 | B. suis | Q933T2 | Q933T2_BRUOV | **97.08** |
| 4 | B. canis | P0A3U5 | OM31_BRUSU | **100.00** |
| 5 | B. ovis | Q71RI3 | Q71RI3_BRUNE | **100.00** |
| 6 | B. ovis | Q93E87 | Q93E87_BRUCA | **99.58** |

1. **LptE Results**

| **Serial number** | **Species** | **UniProt ID-** | **UniProt ID -Species** | **Sequence identity (%)** |
| --- | --- | --- | --- | --- |
| 1 | **B. melitensis** | C0RF57 | C0RF57_BRUMB | **100.00** |
| 2 | B. abortus | Q57B81 | Q57B81_BRUAB | **100.00** |
| 3 | B. suis | A0AAI8H702 | A0AAI8H702_BRUSS | **100.00** |
| 4 | B. canis | A9M844 | A9M844_BRUC2 | **100.00** |
| 5 | B. neotomaez | A0A7U8KBY9 | A0A7U8KBY9_BRUNE | **99.48** |

***Note****: B. canis sequences were not retrieved from UniProt. The analysis compared sequence identity between B. melitensis and* ***four other pathogenic Brucella species****.***pathogenic *Brucella* species**.

1. **VIRB2 Results**

| **Serial number** | **Species** | **UniProt ID-** | **UniProt ID -Species** | **Sequence identity (%)** |
| --- | --- | --- | --- | --- |
| 1: | **B. melitensis** | C0RK20 | C0RK20_BRUMB | **100.00** |
| 2: | B. abortus | P0C528 | VIRB2_BRUAB | **100.00** |
| 3: | B. suis | Q7CEG0 | VIRB2_BRUSU | **100.00** |
| 4: | B. canis | A9MDI5 | A9MDI5_BRUC2 | **100.00** |
| 5: | B. neotomaez | A0A7U8K7C8 | A0A7U8K7C8_BRUNE | **100.00** |

**Note**: *B. ovis* sequences were unavailable in UniProt. Sequence identity was compared between *B. melitensis* and **four other pathogenic *Brucella* species** (*B. abortus*, *B. suis*, *B. canis*, *B. neotomae*).
