# Supplementary material for: Design of a multi-epitope recombinant BCG vaccine targeting Brucella OMP31, LptE and VirB2 in immunoinformatics approaches
Source: PLoS One. 2025 Nov 6;20(11):e0334843. doi: 10.1371/journal.pone.0334843 (PMC12591482; doi:10.1371/journal.pone.0334843)
Supplement: S2 Table — (DOCX) [file pone.0334843.s002.docx]

**S1 Table. MHC-I binding prediction results of OMP31 (IEDB).**

| **allele** | **seq_num** | **start** | **end** | **length** | **peptide** | **core** | **icore** | **score** | **rank** |
| --- | --- | --- | --- | --- | --- | --- | --- | --- | --- |
| HLA-A*11:01 | 1 | 128 | 136 | 9 | AINNNWTLK | AINNNWTLK | AINNNWTLK | 0.824393 | 0.06 |
| HLA-A*03:01 | 1 | 128 | 136 | 9 | AINNNWTLK | AINNNWTLK | AINNNWTLK | 0.820779 | 0.07 |
| HLA-A*11:01 | 1 | 86 | 94 | 9 | GTGGLAYGK | GTGGLAYGK | GTGGLAYGK | 0.752583 | 0.1 |
| HLA-A*03:01 | 1 | 165 | 173 | 9 | TVRVGLNYK | TVRVGLNYK | TVRVGLNYK | 0.641904 | 0.21 |
| HLA-A*02:01 | 1 | 20 | 28 | 9 | VQAGYNWQL | VQAGYNWQL | VQAGYNWQL | 0.603068 | 0.19 |
| HLA-A*11:01 | 1 | 165 | 173 | 9 | TVRVGLNYK | TVRVGLNYK | TVRVGLNYK | 0.554969 | 0.25 |
| HLA-A*03:01 | 1 | 86 | 94 | 9 | GTGGLAYGK | GTGGLAYGK | GTGGLAYGK | 0.379618 | 0.52 |
| HLA-A*02:01 | 1 | 160 | 168 | 9 | KVNFHTVRV | KVNFHTVRV | KVNFHTVRV | 0.359078 | 0.43 |
| HLA-A*11:01 | 1 | 164 | 172 | 9 | HTVRVGLNY | HTVRVGLNY | HTVRVGLNY | 0.351664 | 0.5 |
| HLA-A*02:01 | 1 | 127 | 135 | 9 | YAINNNWTL | YAINNNWTL | YAINNNWTL | 0.311366 | 0.53 |
