# Supplementary material for: Design of a multi-epitope recombinant BCG vaccine targeting Brucella OMP31, LptE and VirB2 in immunoinformatics approaches
Source: PLoS One. 2025 Nov 6;20(11):e0334843. doi: 10.1371/journal.pone.0334843 (PMC12591482; doi:10.1371/journal.pone.0334843)
Supplement: S3 Table — (DOCX) [file pone.0334843.s003.docx]

**S2 Table. MHC-I binding prediction results of LptE (IEDB).**

| **allele** | **seq_num** | **start** | **end** | **length** | **peptide** | **core** | **icore** | **score** | **rank** |
| --- | --- | --- | --- | --- | --- | --- | --- | --- | --- |
| HLA-A*11:01 | 1 | 11 | 19 | 9 | SVTPDMRTK | SVTPDMRTK | SVTPDMRTK | 0.8525 | 0.05 |
| HLA-A*03:01 | 1 | 143 | 151 | 9 | QVYLAVALK | QVYLAVALK | QVYLAVALK | 0.818535 | 0.07 |
| HLA-A*11:01 | 1 | 143 | 151 | 9 | QVYLAVALK | QVYLAVALK | QVYLAVALK | 0.764219 | 0.1 |
| HLA-A*03:01 | 1 | 11 | 19 | 9 | SVTPDMRTK | SVTPDMRTK | SVTPDMRTK | 0.689994 | 0.17 |
| HLA-A*11:01 | 1 | 92 | 100 | 9 | TSNFVLRDK | TSNFVLRDK | TSNFVLRDK | 0.321386 | 0.56 |
| HLA-A*11:01 | 1 | 90 | 98 | 9 | KATSNFVLR | KATSNFVLR | KATSNFVLR | 0.292403 | 0.61 |
| HLA-A*03:01 | 1 | 92 | 100 | 9 | TSNFVLRDK | TSNFVLRDK | TSNFVLRDK | 0.170648 | 1.1 |
| HLA-A*11:01 | 1 | 146 | 154 | 9 | LAVALKMSK | LAVALKMSK | LAVALKMSK | 0.147453 | 1.1 |
| HLA-A*02:01 | 1 | 61 | 69 | 9 | GLSTNTIAA | GLSTNTIAA | GLSTNTIAA | 0.112779 | 1.3 |
| HLA-A*02:01 | 1 | 59 | 67 | 9 | SLGLSTNTI | SLGLSTNTI | SLGLSTNTI | 0.11054 | 1.3 |
