# Supplementary material for: Design of a multi-epitope recombinant BCG vaccine targeting Brucella OMP31, LptE and VirB2 in immunoinformatics approaches
Source: PLoS One. 2025 Nov 6;20(11):e0334843. doi: 10.1371/journal.pone.0334843 (PMC12591482; doi:10.1371/journal.pone.0334843)
Supplement: S4 Table — (DOCX) [file pone.0334843.s004.docx]

**S3 Table. MHC-I binding prediction results of VirB2 (IEDB).**

| **allele** | **seq_num** | **start** | **end** | **length** | **peptide** | **core** | **icore** | **score** | **rank** |
| --- | --- | --- | --- | --- | --- | --- | --- | --- | --- |
| HLA-A*02:01 | 1 | 12 | 20 | 9 | KVLDLLSGV | KVLDLLSGV | KVLDLLSGV | 0.886734 | 0.04 |
| HLA-A*02:01 | 1 | 16 | 24 | 9 | LLSGVSITI | LLSGVSITI | LLSGVSITI | 0.66292 | 0.15 |
| HLA-A*02:01 | 1 | 2 | 10 | 9 | GLDKVNTSM | GLDKVNTSM | GLDKVNTSM | 0.602707 | 0.19 |
| HLA-A*02:01 | 1 | 9 | 17 | 9 | SMQKVLDLL | SMQKVLDLL | SMQKVLDLL | 0.369655 | 0.41 |
| HLA-A*11:01 | 1 | 27 | 35 | 9 | IAIIWSGYK | IAIIWSGYK | IAIIWSGYK | 0.245215 | 0.74 |
| HLA-A*02:01 | 1 | 5 | 13 | 9 | KVNTSMQKV | KVNTSMQKV | KVNTSMQKV | 0.240411 | 0.66 |
| HLA-A*02:01 | 1 | 42 | 50 | 9 | RFMDVVPVL | RFMDVVPVL | RFMDVVPVL | 0.229458 | 0.7 |
| HLA-A*02:01 | 1 | 58 | 66 | 9 | AAAEIASYL | AAAEIASYL | AAAEIASYL | 0.134874 | 1.2 |
| HLA-A*03:01 | 1 | 27 | 35 | 9 | IAIIWSGYK | IAIIWSGYK | IAIIWSGYK | 0.133298 | 1.3 |
| HLA-A*02:01 | 1 | 19 | 27 | 9 | GVSITIVTI | GVSITIVTI | GVSITIVTI | 0.085594 | 1.5 |
