# Supplementary material for: Design of a multi-epitope recombinant BCG vaccine targeting Brucella OMP31, LptE and VirB2 in immunoinformatics approaches
Source: PLoS One. 2025 Nov 6;20(11):e0334843. doi: 10.1371/journal.pone.0334843 (PMC12591482; doi:10.1371/journal.pone.0334843)
Supplement: S5 Table — (DOCX) [file pone.0334843.s005.docx]

**S4 Table. MHC-Ⅱ binding prediction results of OMP31(NetMHC-IIpan-4.1).**

| **Pos** | **MHC** | **Peptide** | **Of** | **Core** | **Core_Rel** | **Identity** | **Score_EL** | **%Rank_EL** | **Antigenicity>0.4** | **allergenicity** | **Theoretical pI** | **Instability index <40** | **Grand average of hydropathicity (GRAVY)** | **Toxicity** |
| --- | --- | --- | --- | --- | --- | --- | --- | --- | --- | --- | --- | --- | --- | --- |
| 61 | DRB1_1501 | ETKVEWFGTVRARLG | 3 | VEWFGTVRA | 1 | Sequence | 0.839689 | 0.37 | 0.2322 |  |  |  |  |  |
| 70 | DRB1_0701 | VRARLGYTATERLMV | 4 | LGYTATERL | 1 | Sequence | 0.825859 | 0.39 | 0.7145 | PROBABLE NON-ALLERGEN | 10.74 | 10.85 | 0.093 | Non-Toxin |
| 79 | DRB1_1501 | TERLMVYGTGGLAYG | 3 | LMVYGTGGL | 1 | Sequence | 0.824893 | 0.41 | -0.0070 |  |  |  |  |  |
| 71 | DRB1_0701 | RARLGYTATERLMVY | 3 | LGYTATERL | 1 | Sequence | 0.81326 | 0.43 | 0.5659 | PROBABLE NON-ALLERGEN | 9.98 | 5.83 | -0.273 | Non-Toxin |
| 60 | DRB1_1501 | AETKVEWFGTVRARL | 4 | VEWFGTVRA | 1 | Sequence | 0.765765 | 0.6 | 0.1392 |  |  |  |  |  |
| 69 | DRB1_0701 | TVRARLGYTATERLM | 5 | LGYTATERL | 1 | Sequence | 0.759509 | 0.61 | 0.8055 | PROBABLE ALLERGEN |  |  |  |  |
| 78 | DRB1_1501 | ATERLMVYGTGGLAY | 4 | LMVYGTGGL | 1 | Sequence | 0.737561 | 0.7 | 0.0407 |  |  |  |  |  |
| 59 | DRB1_1501 | KAETKVEWFGTVRAR | 5 | VEWFGTVRA | 1 | Sequence | 0.693086 | 0.86 | 0.238 |  |  |  |  |  |
| 147 | DRB1_0301 | RNLVDVDNSFLESKV | 3 | VDVDNSFLE | 1 | Sequence | 0.610242 | 1.4 | -0.1306 |  |  |  |  |  |
| 124 | DRB1_0701 | GAEYAINNNWTLKSE | 3 | YAINNNWTL | 1 | Sequence | 0.6005 | 1.38 | 1.3109 | PROBABLE NON-ALLERGEN | 4.53 | 9.71 | -0.907 | Non-Toxin |
