# Supplementary material for: Design of a multi-epitope recombinant BCG vaccine targeting Brucella OMP31, LptE and VirB2 in immunoinformatics approaches
Source: PLoS One. 2025 Nov 6;20(11):e0334843. doi: 10.1371/journal.pone.0334843 (PMC12591482; doi:10.1371/journal.pone.0334843)
Supplement: S6 Table — (DOCX) [file pone.0334843.s006.docx]

**S5 Table. MHC-II binding prediction results of LptE(NetMHC-IIpan-4.1). (DOCX)**

| **Pos** | **MHC** | **Peptide** | **Of** | **Core** | **Core_Rel** | **Identity** | **Score_EL** | **%Rank_EL** | **Exp_Bind** | **BindLevel** | **Antigenicity>0.4** | **allergenicity** | **Theoretical pI** | **Instability index <40** | **Grand average of hydropathicity (GRAVY)** | **Toxicity** |
| --- | --- | --- | --- | --- | --- | --- | --- | --- | --- | --- | --- | --- | --- | --- | --- | --- |
| 8 | DRB1_0301 | IGGSVTPDMRTKLAS | 4 | VTPDMRTKL | 1 | Sequence | 0.937527 | 0.12 | NA | <=SB | 0.7282 | PROBABLE NON-ALLERGEN | 8.75 | -3.81 | -0.073 | Non-Toxin |
| 9 | DRB1_0301 | GGSVTPDMRTKLASI | 3 | VTPDMRTKL | 1 | Sequence | 0.937402 | 0.12 | NA | <=SB | 0.8665 | PROBABLE NON-ALLERGEN | 8.75 | -3.81 | -0.073 | Non-Toxin |
| 85 | DRB1_0701 | SAGIVKATSNFVLRD | 3 | IVKATSNFV | 0.767 | Sequence | 0.924749 | 0.12 | NA | <=SB | 0.3359 |  |  |  |  |  |
| 7 | DRB1_0301 | AIGGSVTPDMRTKLA | 5 | VTPDMRTKL | 1 | Sequence | 0.89596 | 0.27 | NA | <=SB | 0.8233 | PROBABLE ALLERGEN |  |  |  |  |
| 86 | DRB1_0701 | AGIVKATSNFVLRDK | 3 | VKATSNFVL | 0.76 | Sequence | 0.887525 | 0.23 | NA | <=SB | 0.0042 |  |  |  |  |  |
| 20 | DRB1_0301 | LASIAIDPAGDIFGQ | 3 | IAIDPAGDI | 1 | Sequence | 0.873049 | 0.34 | NA | <=SB | 0.1516 |  |  |  |  |  |
| 84 | DRB1_0701 | PSAGIVKATSNFVLR | 4 | IVKATSNFV | 0.833 | Sequence | 0.863184 | 0.28 | NA | <=SB | 0.4667 | PROBABLE NON-ALLERGEN | 11.01 | 11.75 | 0.46 | Non-Toxin |
| 19 | DRB1_0301 | KLASIAIDPAGDIFG | 4 | IAIDPAGDI | 1 | Sequence | 0.824398 | 0.5 | NA | <=SB | 0.1931 |  |  |  |  |  |
| 85 | DRB1_1501 | SAGIVKATSNFVLRD | 3 | IVKATSNFV | 0.873 | Sequence | 0.821478 | 0.42 | NA | <=SB | 0.3359 |  |  |  |  |  |
| 10 | DRB1_0301 | GSVTPDMRTKLASIA | 2 | VTPDMRTKL | 1 | Sequence | 0.817281 | 0.53 | NA | <=SB | 0.6384 | PROBABLE NON-ALLERGEN | 8.75 | -12.04 | 0.073 |  |
