# Supplementary material for: Design of a multi-epitope recombinant BCG vaccine targeting Brucella OMP31, LptE and VirB2 in immunoinformatics approaches
Source: PLoS One. 2025 Nov 6;20(11):e0334843. doi: 10.1371/journal.pone.0334843 (PMC12591482; doi:10.1371/journal.pone.0334843)
Supplement: S7 Table — (DOCX) [file pone.0334843.s007.docx]

**S6 Table. MHC-Ⅱ binding prediction results of VirB2(NetMHC-IIpan-4.1). (DOCX)**

| **MHC** | **Peptide** | **Of** | **Core** | **Core_Rel** | **Identity** | **Score_EL** | **%Rank_EL** | **Exp_Bind BindLevel** | **Antigenicity>0.4** | **allergenicity** | **Theoretical pI** | **Instability index<40** | **Grand average of hydropathicity (GRAVY)** | **Toxicity** |
| --- | --- | --- | --- | --- | --- | --- | --- | --- | --- | --- | --- | --- | --- | --- |
| DRB1_0701 | LGGALVVGAAAEIAS | 4 | LVVGAAAEI | 0.88 | Sequence | 0.268989 | 5.07 | NA | 0.5069 | PROBABLE NON-ALLERGEN | 4 | 13.42 | 1.6 | Non-Toxin |
| DRB1_0701 | GGALVVGAAAEIASY | 3 | LVVGAAAEI | 0.807 | Sequence | 0.261351 | 5.26 | NA | 0.4919 | PROBABLE NON-ALLERGEN | 4 | 13.42 | 1.26 | Non-Toxin |
| DRB1_0701 | YKMAFRHARFMDVVP | 4 | FRHARFMDV | 0.98 | Sequence | 0.217018 | 6.4 | NA | 0.8614 | PROBABLE NON-ALLERGEN | 9.99 | 64.97 | -0.073 | Non-Toxin |
| DRB1_0701 | KMAFRHARFMDVVPV | 3 | FRHARFMDV | 0.967 | Sequence | 0.215637 | 6.44 | NA | 0.811 | PROBABLE NON-ALLERGEN | 10.84 | 77.81 | 0.293 | Non-Toxin |
| DRB1_1501 | VTIAIIWSGYKMAFR | 3 | AIIWSGYKM | 0.9 | Sequence | 0.190059 | 6.63 | NA | 0.3253 |  |  |  |  |  |
| DRB1_0701 | RHARFMDVVPVLGGA | 4 | FMDVVPVLG | 0.933 | Sequence | 0.182902 | 7.59 | NA | 0.5492 | PROBABLE NON-ALLERGEN | 9.61 | 50.42 | 0.44 | Non-Toxin |
| DRB1_0701 | VLGGALVVGAAAEIA | 5 | LVVGAAAEI | 0.913 | Sequence | 0.16423 | 8.39 | NA | 0.5320 | PROBABLE NON-ALLERGEN | 4 | 13.42 | 1.933 | Non-Toxin |
