# Supplementary material for: Design of a multi-epitope recombinant BCG vaccine targeting Brucella OMP31, LptE and VirB2 in immunoinformatics approaches
Source: PLoS One. 2025 Nov 6;20(11):e0334843. doi: 10.1371/journal.pone.0334843 (PMC12591482; doi:10.1371/journal.pone.0334843)
Supplement: S8 Table — (DOCX) [file pone.0334843.s008.docx]

**S7 Table. LBEs results of OMP31(ABCpred and IEDB).**

| **Rank** | **Sequence** | **Start position** | **Score** | **Antigenicity >0.4** | **Allergenicity** | **Theoretical pI** | **Instability index <40** | **Grand average of hydropathicity (GRAVY)** | **Toxicity** |
| --- | --- | --- | --- | --- | --- | --- | --- | --- | --- |
| 1 | LHTWSDKTKAGWTLGA | 108 | 0.9 | 0.8622 | PROBABLE NON-ALLERGEN | 8.6 | -17.03 | -0.55 | Non-Toxin |
| 2 | GWTLGAGAEYAINNNW | 118 | 0.89 | 1.0419 | PROBABLE NON-ALLERGEN | 4 | 5.4 | -0.331 | Non-Toxin |
| 3 | TGSISAGASGLEGKAE | 46 | 0.88 | 1.6736 | PROBABLE NON-ALLERGEN | 4.53 | -6.54 | -0.119 | Non-Toxin |
| 4 | KVEWFGTVRARLGYTA | 63 | 0.84 | 0.2833 |  |  |  |  |  |
| 5 | MVYGTGGLAYGKVKSA | 83 | 0.79 | 0.4516 | PROBAB  E NON-ALLERGEN | 9.52 | -21.26 | 0.263 | Non-Toxin |

1. **ABCpred predicion result**

**2. IEBD predicion result**

|  |
| --- |

| **No.** | **Start** | **End** | **Peptide** | **Length** | **Rank** |
| --- | --- | --- | --- | --- | --- |
| 1 | 4 | 14 | QVSGSLDVTAG | 11 | 2 |
| 2 | 24 | 30 | YNWQLDN | 7 | 3 |
| 3 | 40 | 63 | FQGSSVTGSISAGASGLEGKAETK | 24 | 2 |
| 4 | 72 | 80 | ARLGYTATE | 9 | 3 |
| 5 | 93 | 116 | GKVKSAFNLGDDASALHTWSDKTK | 24 | 1 |
| 6 | 129 | 164 | INNNWTLKSEYLYTDLGKRNLVDVDNSFLESKVNFH | 36 | 2 |
| 7 | 170 | 170 | L | 1 | 3 |

|  |
| --- |

|  |
| --- |
