# Supplementary material for: Design of a multi-epitope recombinant BCG vaccine targeting Brucella OMP31, LptE and VirB2 in immunoinformatics approaches
Source: PLoS One. 2025 Nov 6;20(11):e0334843. doi: 10.1371/journal.pone.0334843 (PMC12591482; doi:10.1371/journal.pone.0334843)
Supplement: S9 Table — (DOCX) [file pone.0334843.s009.docx]

**S8 Table. LBEs results of LptE (ABCpred and IEDB).**

1. **ABCpred predicion result**

| **Rank** | **Sequence** | **Start position** | **Score** | **Antigenicity >0.4** | **Allergenicity** | **Theoretical pI** | **Instability index <40** | **Grand average of hydropathicity (GRAVY)** | **Toxicity** |
| --- | --- | --- | --- | --- | --- | --- | --- | --- | --- |
| 1 | GSAIGGSVTPDMRTKL | 5 | 0.9 | 0.9958 | PROBABLE NON-ALLERGEN | 8.75 | -2.95 | -0.094 | Non-Toxin |
| 2 | TGSRMVAASFDRPRQE | 107 | 0.89 | 0.2579 |  |  |  |  |  |
| 3 | DIGDQTDRTGRPSAGI | 73 | 0.87 | 1.2370 | PROBABLE NON-ALLERGEN | 4.43 | 18.12 | -1.075 | Non-Toxin |
| 4 | TNTIAAVSVDIGDQTD | 64 | 0.81 | 0.5291 | PROBABLE NON-ALLERGEN | 3.42 | -14.72 | 0.013 | Non-Toxin |
| 4 | AGDIFGQEVRNELIFL | 28 | 0.81 | 0.3859 |  |  |  |  |  |
| 5 | AGIVKATSNFVLRDKD | 86 | 0.8 | 0.0633 |  |  |  |  |  |

**2. IEBD predicion result**

| **No.** | **Start** | **End** | **Peptide** | **Length** |
| --- | --- | --- | --- | --- |
| 1 | 5 | 23 | GSAIGGSVTPDMRTKLASI | 19 |
| 2 | 28 | 28 | A | 1 |
| 3 | 30 | 31 | DI | 2 |
| 4 | 44 | 55 | FSGGAGEPANPA | 12 |
| 5 | 66 | 86 | TIAAVSVDIGDQTDRTGRPSA | 21 |
| 6 | 98 | 107 | RDKDGKPLAT | 10 |
| 7 | 114 | 126 | ASFDRPRQEFANL | 13 |
