# Supplementary material for: Design of a multi-epitope recombinant BCG vaccine targeting Brucella OMP31, LptE and VirB2 in immunoinformatics approaches
Source: PLoS One. 2025 Nov 6;20(11):e0334843. doi: 10.1371/journal.pone.0334843 (PMC12591482; doi:10.1371/journal.pone.0334843)
Supplement: S10 Table — (DOCX) [file pone.0334843.s010.docx]

**S9 Table. LBEs results of VirB2 (ABCpred and IEDB).**

1. **ABCpred predicion result**

| **Rank** | **Sequence** | **Start position** | **Score** | **Antigenicity >0.4** | **Allergenicity** | **Theoretical pI** | **Instability index <40** | **Grand average of hydropathicity (GRAVY)** | **Toxicity** |
| --- | --- | --- | --- | --- | --- | --- | --- | --- | --- |
| 1 | TIAIIWSGYKMAFRHA | 26 | 0.84 | 0.0692 |  |  |  |  |  |
| 2 | VPVLGGALVVGAAAEI | 47 | 0.73 | 0.5779 |  | 4.00 | 37.28 | 1.863 | Non-Toxin |
| 3 | YKMAFRHARFMDVVPV | 34 | 0.68 | 0.8455 | PROBABLE ALLERGEN |  |  |  |  |
| 4 | GGLDKVNTSMQKVLDL | 1 | 0.6 | 0.5398 | PROBABLE NON-ALLERGEN | 5.96 | -8.85 | -0.15 | Non-Toxin |
| 5 | ALVVGAAAEIASYLLR | 53 | 0.53 | 0.4778 |  | 6.05 | 22.84 | 1.485 | Non-Toxin |

1. **IEBD predicion result**

| **No.** | **Start** | **End** | **Peptide** | **Length** | **Rank** |
| --- | --- | --- | --- | --- | --- |
| 1 | 8 | 8 | T | 1 | 2 |
| 2 | 11 | 11 | Q | 1 | 2 |
| 3 | 39 | 45 | RHARFMD | 7 | 1 |
