# Supplementary material for: Design of a multi-epitope recombinant BCG vaccine targeting Brucella OMP31, LptE and VirB2 in immunoinformatics approaches
Source: PLoS One. 2025 Nov 6;20(11):e0334843. doi: 10.1371/journal.pone.0334843 (PMC12591482; doi:10.1371/journal.pone.0334843)
Supplement: S11 Table — (DOCX) [file pone.0334843.s011.docx]

| **No** | **Residues** | **Number of residues** | **Score** | **3D structure** | **Antigenicity >0.4** | **allergenicity** | **Theoretical pI** | **Instability index <40** | **Grand average of hydropathicity (GRAVY)** | **Toxicity** |
| --- | --- | --- | --- | --- | --- | --- | --- | --- | --- | --- |
| 1 | SVILASIAAMF | 11 | 0.966 |  | 0.2017 |  |  |  |  |  |
| 2 | KPFSSFDKEDNEQVSGSLDNSFE | 23 | 0.785 |  | 0.5306 | PROBABLE NON-ALLERGEN | 4.02 | 38.18 | -1.257 | Non-Toxin |
| 3 | ATSAMAADVVVSEPSA | 16 | 0.777 |  | 0.7549 | PROBABLE NON-ALLERGEN | 3.67 | 47.89 | 0.738 | Non-Toxin |
| 4 | EANLGDDASALHTW | 14 | 0.749 |  | 0.6442 | PROBABLE NON-ALLERGEN | 4.02 | -6.84 | -0.5 | Non-Toxin |
| 5 | SAGASGLEGK | 10 | 0.746 |  | 2.3499 | PROBABLE NON-ALLERGEN | 5.72 | -7.98 | -0.28 | Non-Toxin |

**S10 Table. CBEs results of OMP31 (IEDB).**
