# Supplementary material for: Design of a multi-epitope recombinant BCG vaccine targeting Brucella OMP31, LptE and VirB2 in immunoinformatics approaches
Source: PLoS One. 2025 Nov 6;20(11):e0334843. doi: 10.1371/journal.pone.0334843 (PMC12591482; doi:10.1371/journal.pone.0334843)
Supplement: S12 Table — (DOCX) [file pone.0334843.s012.docx]

**S11 Table. CBEs results of LptE (IEDB).**

| **No.** | **Residues** | **Number of residues** | **Score** | **3D structure** | **Antigenicity >0.4** | **Allergenicity** | **Theoretical pI** | **Instability index <40** | **Grand average of hydropathicity (GRAVY)** | **Toxicity** |
| --- | --- | --- | --- | --- | --- | --- | --- | --- | --- | --- |
| 1 | SLPD | 4 | 0.982 |  | - | - | - | - | - | - |
| 2 | LSAIKAFCIGFFALGAAVLI | 20 | 0.798 |  | 0.3457 |  |  |  |  |  |
| 3 | AAVSVDIGDQTDTGRPSADRPQEFANLR | 28 | 0.787 |  | 0.4913 | PROBABLE NON-ALLERGEN | 4.36 | 31.42 | -0.779 | Non-Toxin |
| 4 | SGGAGEPANPAYRDKDGKPL | 20 | 0.683 |  | 1.1114 | PROBABLE NON-ALLERGEN | 5.84 | 14.39 | -1.28 | Non-Toxin |
| 5 | SSNHGAGSAIGGSVTPDT | 18 | 0.542 |  | 1.4803 | PROBABLE NON-ALLERGEN | 5.06 | -0.73 | -0.317 | Non-Toxin |
