# Supplementary material for: Design of a multi-epitope recombinant BCG vaccine targeting Brucella OMP31, LptE and VirB2 in immunoinformatics approaches
Source: PLoS One. 2025 Nov 6;20(11):e0334843. doi: 10.1371/journal.pone.0334843 (PMC12591482; doi:10.1371/journal.pone.0334843)
Supplement: S13 Table — (DOCX) [file pone.0334843.s013.docx]

**S12 Table. CBEs results of VirB2 (IEDB).**

| **No.** | **Residues** | **Number of residues** | **Score** | **3D structure** | **Antigenicity >0.4** | **Allergenicity** | **Theoretical pI** | **Instability index <40** | **Grand average of hydropathicity (GRAVY)** | **Toxicity** |
| --- | --- | --- | --- | --- | --- | --- | --- | --- | --- | --- |
| 1 | KTASPSKKSLSRILPH | 16 | 0.82 |  | 0.3519 |  |  |  |  |  |
| 2 | ANGGLDKVNT | 10 | 0.703 |  | 0.6563 | PROBABLE ALLERGEN |  |  |  |  |
| 3 | GYKMAFRARFMDVV | 14 | 0.699 |  | 1.0409 | PROBABLE NON-ALLERGEN | 9.99 | 35.32 | 0.236 | Non-Toxin |
| 4 | EPNAH | 5 | 0.619 |  | / | PROBABLE ALLERGEN |  |  |  |  |
