# Supplementary material for: Design of a multi-epitope recombinant BCG vaccine targeting Brucella OMP31, LptE and VirB2 in immunoinformatics approaches
Source: PLoS One. 2025 Nov 6;20(11):e0334843. doi: 10.1371/journal.pone.0334843 (PMC12591482; doi:10.1371/journal.pone.0334843)
Supplement: S14 Table — (DOCX) [file pone.0334843.s014.docx]

**S13 Table. Tertiary structure results of multi-epitope vaccines optimised by GalaxyRefine.**

| **Model** | **GDT-HA** | **RMSD** | **MolProbity** | **Clash score** | **Poor rotamers** | **Rama favored** |
| --- | --- | --- | --- | --- | --- | --- |
| Initial | 1.0000 | 0.000 | 1.536 | 3.1 | 0.0 | 93.4 |
| MODEL 1 | 0.9887 | 0.306 | 1.942 | 10.1 | 0.0 | 93.7 |
| MODEL 2 | 0.9847 | 0.317 | 1.976 | 11.7 | 0.7 | 94.2 |
| MODEL 3 | 0.9783 | 0.341 | 1.973 | 11.2 | 0.4 | 93.9 |
| MODEL 4 | 0.9871 | 0.304 | 1.934 | 10.3 | 0.2 | 94.0 |
| MODEL 5 | 0.9827 | 0.322 | 1.977 | 11.3 | 0.7 | 93.9 |
