# Supplementary material for: Design of a multi-epitope recombinant BCG vaccine targeting Brucella OMP31, LptE and VirB2 in immunoinformatics approaches
Source: PLoS One. 2025 Nov 6;20(11):e0334843. doi: 10.1371/journal.pone.0334843 (PMC12591482; doi:10.1371/journal.pone.0334843)
Supplement: S15 Table — (DOCX) [file pone.0334843.s015.docx]

**S14 Table. Screening of disulfide bonds by using the Disulfide by Design server.**

| **Disulfide by Design** | **2.13** |  |  |  |  |  |  |  |
| --- | --- | --- | --- | --- | --- | --- | --- | --- |
| **Input File** | **model_2.pdb** |  |  |  |  |  |  |  |
| **Input File Model** | **1** |  |  |  |  |  |  |  |
| **Number of complete residues** | **621** |  |  |  |  |  |  |  |
| **Optimum Chi3 angle: +97/-87 with Tolerance** | **30** |  |  |  |  |  |  |  |
| **Optimum Ca-Cb-S angle: 114.60 with Tolerance** | **10** |  |  |  |  |  |  |  |
| **Check for INTER-chain disulfides** | **TRUE** |  |  |  |  |  |  |  |
| **Check for INTRA-chain disulfides** | **TRUE** |  |  |  |  |  |  |  |
|  |  |  |  |  |  |  |  |  |
| Res1 Chain | Res1 Seq # | Res1 AA | Res2 Chain | Res2 Seq # | Res2 AA | Chi3 | Energy | Sum B-Factors |
| A | 3 | PRO | A | 8 | ASP | -81.45 | 2.21 | 0 |
| A | 6 | ILE | A | 85 | LYS | 109.96 | 1.38 | 0 |
| A | 23 | ASP | A | 44 | LYS | 105.52 | 3.32 | 0 |
| A | 28 | TYR | A | 77 | TYR | -87.75 | 0.82 | 0 |
| A | 39 | ALA | A | 51 | VAL | 97.63 | 4.68 | 0 |
| A | 58 | HIS | A | 62 | GLN | 121.34 | 5.89 | 0 |
| A | 82 | LYS | A | 103 | ALA | -97.95 | 5.78 | 0 |
| A | 84 | GLU | A | 103 | ALA | 102.21 | 3.37 | 0 |
| A | 85 | LYS | A | 101 | SER | 118.73 | 5.4 | 0 |
| A | 87 | CYS | A | 98 | ALA | -104.59 | 3.52 | 0 |
| A | 87 | CYS | A | 99 | ALA | 93.25 | 4.01 | 0 |
| A | 104 | ASN | A | 107 | ALA | 120.24 | 2.23 | 0 |
| A | 114 | ALA | A | 139 | ALA | 103.15 | 1.4 | 0 |
| A | 117 | LEU | A | 132 | GLY | 73.21 | 4.6 | 0 |
| A | 122 | ALA | A | 153 | VAL | 108.02 | 4.33 | 0 |
| A | 122 | ALA | A | 154 | GLY | -72.14 | 5.74 | 0 |
| A | 158 | ALA | A | 170 | ALA | 81.71 | 0.95 | 0 |
| A | 158 | ALA | A | 171 | ALA | -110.86 | 3.13 | 0 |
| A | 163 | THR | A | 166 | MET | 115.43 | 1.89 | 0 |
| A | 172 | TYR | A | 337 | PRO | -105.86 | 3.45 | 0 |
| A | 175 | THR | A | 341 | ALA | 105.33 | 2.73 | 0 |
| A | 178 | PHE | A | 341 | ALA | 79.23 | 5.8 | 0 |
| A | 182 | ALA | A | 348 | ALA | 95.63 | 3.36 | 0 |
| A | 189 | LEU | A | 352 | SER | 90.95 | 4.2 | 0 |
| A | 190 | LYS | A | 321 | GLY | 97.91 | 3.56 | 0 |
| A | 194 | GLY | A | 317 | PRO | 76.96 | 3.5 | 0 |
| A | 209 | GLU | A | 226 | ALA | 92.67 | 2.69 | 0 |
| A | 218 | GLY | A | 257 | PRO | 92.25 | 3.98 | 0 |
| A | 219 | ARG | A | 236 | GLY | -104.91 | 3.45 | 0 |
| A | 221 | ARG | A | 232 | VAL | 95.77 | 0.14 | 0 |
| A | 223 | GLY | A | 230 | LEU | 107.59 | 3.34 | 0 |
| A | 223 | GLY | A | 232 | VAL | -97.78 | 5.52 | 0 |
| A | 241 | GLU | A | 251 | LYS | 98.54 | 7.61 | 0 |
| A | 243 | ALA | A | 249 | THR | 98.8 | 2.6 | 0 |
| A | 252 | SER | A | 256 | GLY | 77.63 | 6.1 | 0 |
| A | 258 | GLY | A | 283 | THR | 88.17 | 2.84 | 0 |
| A | 260 | GLY | A | 283 | THR | 80.32 | 3.53 | 0 |
| A | 276 | GLY | A | 280 | GLY | 82.26 | 4.05 | 0 |
| A | 277 | PRO | A | 280 | GLY | -75.37 | 4.3 | 0 |
| A | 309 | ASN | A | 329 | ALA | 91.67 | 1.46 | 0 |
| A | 313 | ARG | A | 323 | LEU | 124.9 | 3.45 | 0 |
| A | 320 | GLY | A | 359 | VAL | -109.55 | 2.38 | 0 |
| A | 321 | GLY | A | 351 | ALA | 102.23 | 4.28 | 0 |
| A | 324 | VAL | A | 351 | ALA | 106.33 | 3.09 | 0 |
| A | 324 | VAL | A | 362 | GLY | 81.48 | 3.67 | 0 |
| A | 328 | ALA | A | 347 | ALA | -78.37 | 1.03 | 0 |
| A | 331 | ILE | A | 369 | ALA | -100.41 | 1.03 | 0 |
| A | 332 | ALA | A | 343 | VAL | 122.56 | 5.18 | 0 |
| A | 332 | ALA | A | 373 | ALA | -87.09 | 0.29 | 0 |
| A | 338 | GLY | A | 377 | HIS | 112.57 | 3.68 | 0 |
| A | 347 | ALA | A | 365 | VAL | 112.1 | 3.64 | 0 |
| A | 350 | ILE | A | 361 | GLY | 119.18 | 4.23 | 0 |
| A | 353 | TYR | A | 358 | GLY | -58.24 | 4.85 | 0 |
| A | 396 | THR | A | 418 | GLY | 109.06 | 3.73 | 0 |
| A | 398 | GLY | A | 416 | SER | -77.16 | 3.76 | 0 |
| A | 398 | GLY | A | 469 | ILE | -89.9 | 3.96 | 0 |
| A | 401 | ALA | A | 473 | SER | 104.8 | 2.66 | 0 |
| A | 418 | GLY | A | 432 | ALA | -94.09 | 3.82 | 0 |
| A | 420 | SER | A | 429 | LYS | 94.08 | 5.99 | 0 |
| A | 432 | ALA | A | 466 | THR | -110.15 | 3.58 | 0 |
| A | 479 | GLN | A | 551 | ASP | 117 | 4.69 | 0 |
| A | 498 | ASP | A | 515 | VAL | -80.59 | 2.45 | 0 |
| A | 530 | LEU | A | 533 | ASP | -81.34 | 5.52 | 0 |
| A | 543 | ALA | A | 607 | VAL | 124.28 | 4.54 | 0 |
| A | 543 | ALA | A | 608 | VAL | -71.17 | 3.6 | 0 |
| A | 546 | SER | A | 601 | ARG | -85.63 | 1.36 | 0 |
| A | 560 | ALA | A | 594 | LYS | 91.85 | 4.99 | 0 |
| A | 575 | GLY | A | 578 | GLU | 118.63 | 5.73 | 0 |
