# Supplementary figures and images for: Design of a multi-epitope recombinant BCG vaccine targeting Brucella OMP31, LptE and VirB2 in immunoinformatics approaches
Source: PLoS One. 2025 Nov 6;20(11):e0334843. doi: 10.1371/journal.pone.0334843 (PMC12591482; doi:10.1371/journal.pone.0334843)

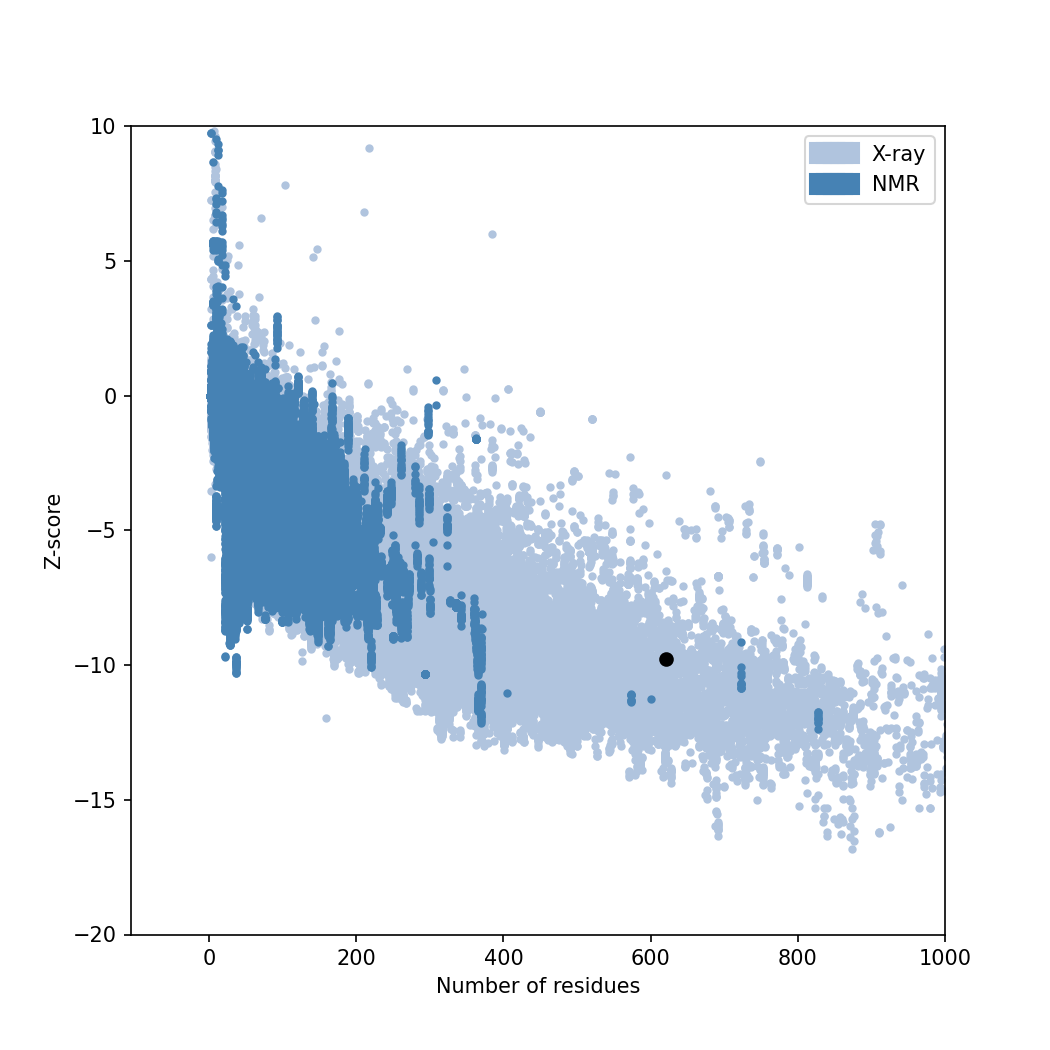

Supplement: S1 Fig — (TIF) [file pone.0334843.s016.tif]

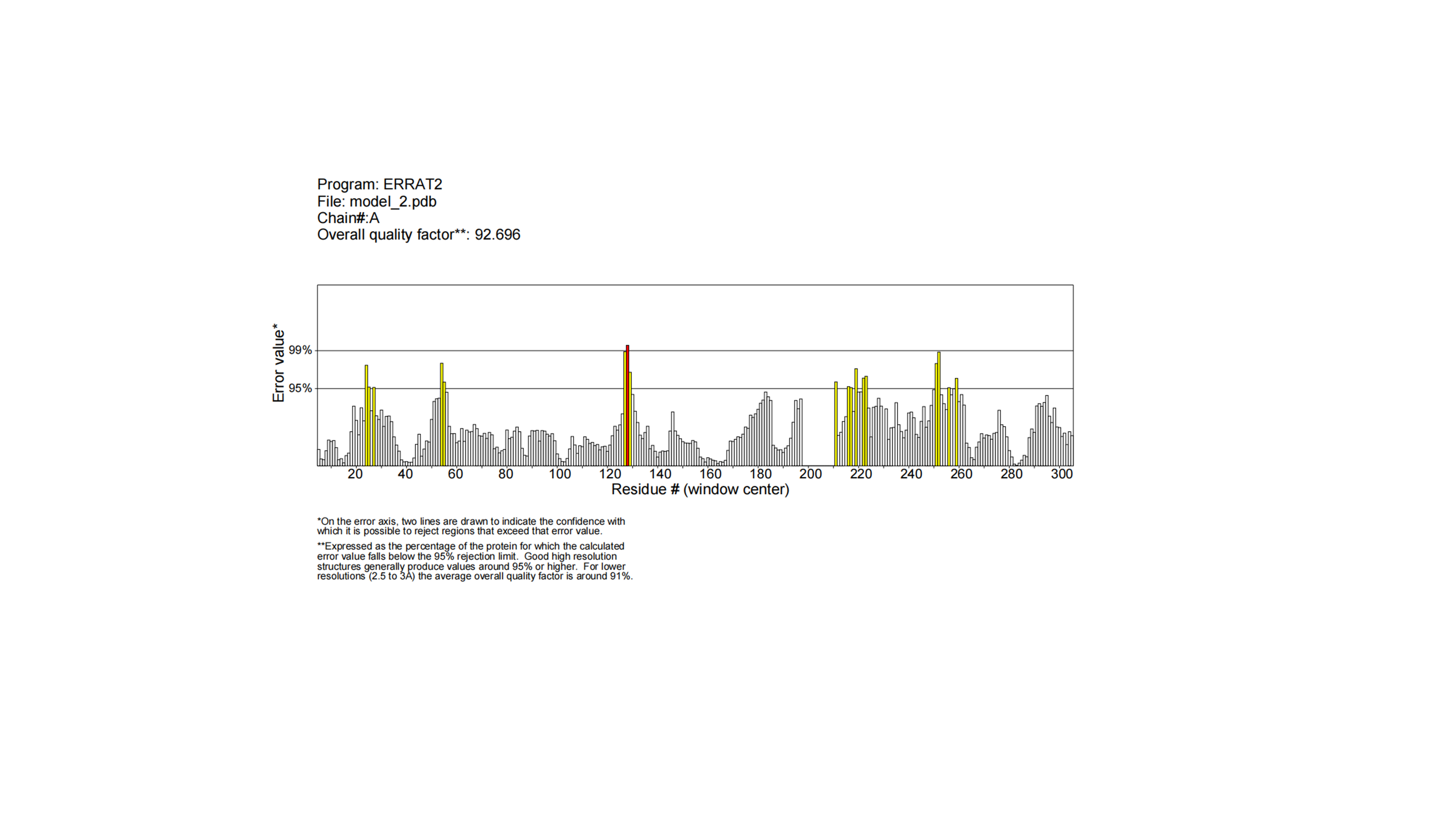

Supplement: S2 Fig — (TIF) [file pone.0334843.s017.tif]

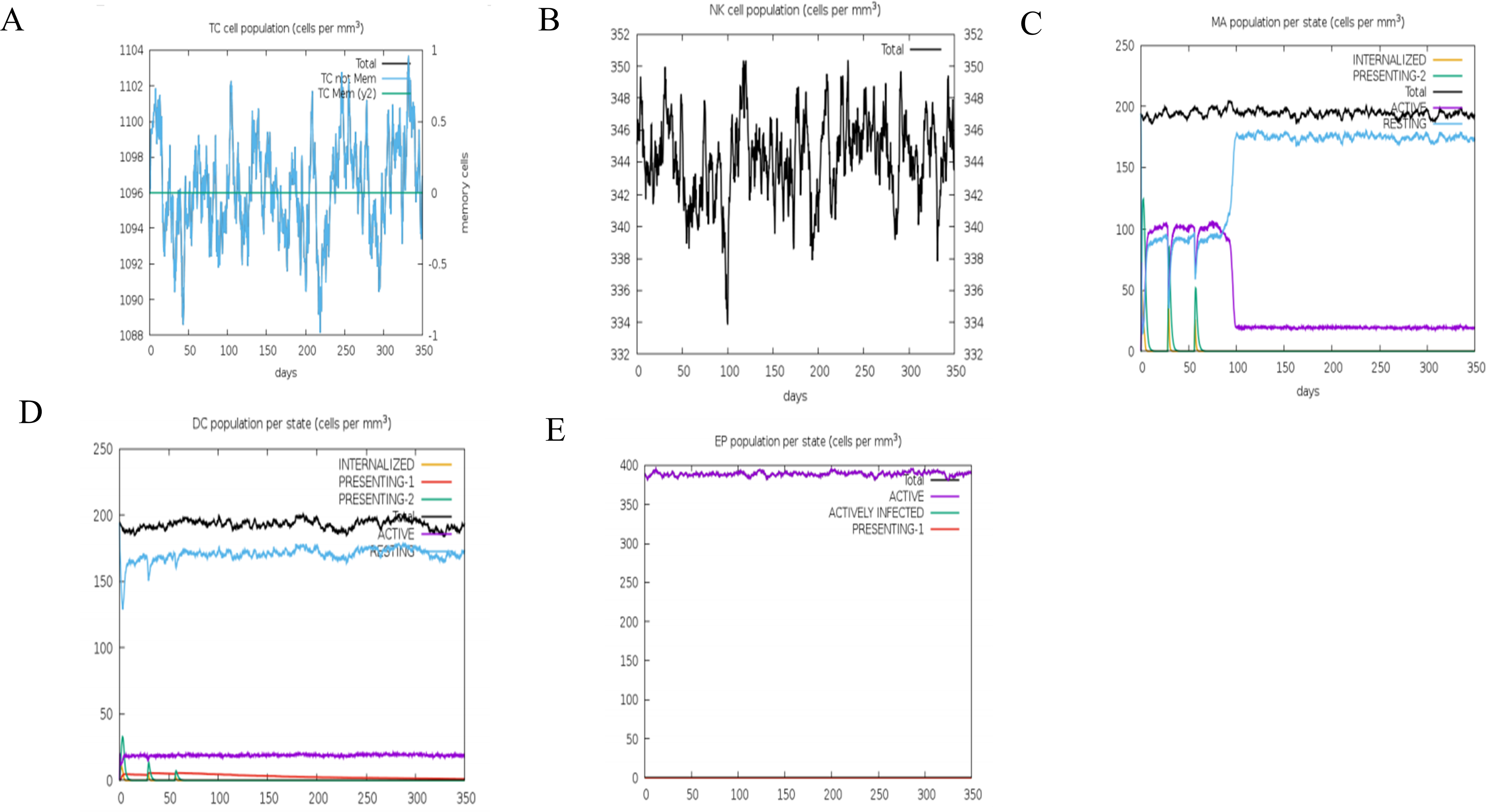

Supplement: S3 Fig — (TIF) [file pone.0334843.s018.tif]
